# Polycyclic aromatic hydrocarbons exposure as a potential risk factor for miscarriage among women in the United States: A secondary dataset analysis of NHANES data for the period 2005–2014

**DOI:** 10.18332/tid/205903

**Published:** 2025-08-08

**Authors:** Xiaoxing Liu, Yanmei Li, Na Chen, Jianshuang Ma, Yujuan Xing, Fengxia Miao

**Affiliations:** 1 Department of Obstetrics, Dongying People's Hospital, Dongying, China; 2 Department of Obstetrics, Guangrao People's Hospital, Dongying, China; 3 Department of Gynecology, Dongying People's Hospital, Dongying, China

**Keywords:** polycyclic aromatic hydrocarbons, miscarriage, tobacco, National Health and Nutrition Examination Survey

## Abstract

**INTRODUCTION:**

Numerous studies have shown that polycyclic aromatic hydrocarbons (PAHs) are endocrine disruptors associated with reproduction, with tobacco smoke identified as a major non-occupational source of PAH exposure. However, there is still a lack of information on the relationship between PAH exposure – particularly from tobacco-related sources – and miscarriage.

**METHODS:**

The data for this study were obtained from the National Health and Nutrition Examination Survey (NHANES) 2005–2014. Excluding populations with missing PAH, miscarriage, or baseline information, a total of 2573 individuals were included in this study. Logistic regression, linear regression, restricted cubic spline (RCS) analysis and subgroup analysis were used to analyze the effects of PAHs.

**RESULTS:**

Following logistic and linear regression analyses, we found that higher concentrations of 2-hydroxynaphthalene, 3-hydroxyfluorene, 2-hydroxyfluorene, 1-hydroxyphenanthrene, and 1-hydroxypyrene were associated with miscarriage (p<0.05, OR>1). Moreover, after RCS, we found a nonlinear relationship between 1-hydroxynaphthalene and miscarriage (p=0.01). The relationship between 1-hydroxynaphthalene and miscarriage could be described as an ‘n-shaped’ curve, with a cutoff value (4705 ng/L). At concentrations lower than the cutoff, there was a positive correlation between 1-hydroxynaphthalene and miscarriage. Conversely, at concentrations higher than the cutoff, there was a negative correlation between the two variables. Finally, a subgroup analysis was performed to explore the interaction effect of confounders with the outcome variables, to further demonstrate the robustness of the results.

**CONCLUSIONS:**

The probability of miscarriage increases with increasing concentration of certain PAHs in the body. Enhancing monitoring of tobacco-related PAHs exposure is highly important for the prevention of miscarriage.

## INTRODUCTION

During the incomplete burning of coal, oil and gas, garbage, and other organic substances, polycyclic aromatic hydrocarbons (PAHs) are produced^1^. PAHs are widely present in the environment and food. In terms of human exposure sources, PAHs can be found in vehicle exhaust emissions, asphalt, agricultural burning, burnt food, tobacco smoke, wastewater, marine oil spills, and aquatic products^2,3^. Among these sources, combustible cigarettes are a major contributor to PAHs exposure^4^. For example, a study in Northwestern Ontario’s First Nations communities showed that urinary PAH levels among smokers were significantly higher than those among non-smokers^5^.

Once PAHs enter the human body through various exposure pathways, they undergo hydroxylation and glucuronidation metabolism. In this study, capillary gas chromatography–high resolution mass spectrometry (GC–HRMS) was employed to identify urinary metabolites, specifically monohydroxy-polycyclic aromatic hydrocarbons (OH-PAHs). PAHs are a class of environmental disruptors and are associated with reproductive disorders such as male infertility, breast cancer, and sex hormone levels^6,7^. For example, 2-hydroxynapthalene and 3-hydroxyfluorene are associated with increased serum testosterone levels in men, while 1-hydroxyphenanthrene is associated with increased serum estradiol in women^8^. In addition, increased exposure to mixed PAHs is associated with elevated alanine aminotransferase (ALT) in women and affects the natural age of menopause^9^. It is also positively associated with an increased incidence of coronary heart disease^10^.

It is estimated that 23 million miscarriages occur worldwide each year^11^. The frequency of miscarriage deaths is very high and is greater than the number of deaths from major diseases^12^. Miscarriage is associated with risk factors such as age, BMI, race, smoking, alcohol consumption, type of work, infections and pollutants^13,14^. PAHs were among the pollutants found in our study. Miscarriage not only poses physical risks such as bleeding and infection but also causes psychological burdens that can lead to anxiety and depression and even increase the probability of suicide^11^. Furthermore, its impact extends beyond individuals and families to affect broader societal dimensions. The reported annual cost of miscarriage in the UK is estimated to be £471 million^11^. Therefore, miscarriage is a great burden both for the patients themselves and for the family’s psychological and physiological wellbeing. To reduce the societal burden, identifying various etiologies, risk factors, and treatment measures is necessary. However, no prior studies have explicitly investigated the specific association between PAHs – with tobacco as a major exposure source – and miscarriage. Here, we aimed to examine the association between PAH exposure and miscarriage in a cross-sectional study among women of childbearing age. This study will provide guidance for the prevention, monitoring, and treatment of miscarriage^15^.

## METHODS

### Study population

We extracted the sample from the NHANES database, a cross-sectional study designed to collect representative data on the health and nutritional status of the US population. The dataset includes survey information from a demographically diverse sample of individuals, allowing for population-level analyses. The survey covers a wide range of factors, including chronic conditions, dietary intake, physical activity, and biomarkers. By examining these various aspects, the database provides valuable insights into the health and nutritional status of the population. The collected data can aid in monitoring health trends, identifying disparities, and informing public health policies and programs. To establish the study sample, we extracted data from the 2005–2014 cycles of NHANES, which initially included 50965 participants. We first selected 12863 individuals with available urine samples for PAH metabolite analysis. Participants were then required to have complete responses to the pregnancy-related questionnaire items ‘RHQ160: How many times have you been pregnant?’ and ‘RHQ171: How many deliveries had a live birth result?’; those with missing values for either question were excluded. This selection process resulted in a final eligible sample of 2573 women. The NHANES is conducted by the Centers for Disease Control and Prevention (CDC) and the National Center for Health Statistics (NCHS). The study protocol has been approved by the NCHS Research Ethics Review Committee. The approval numbers can be found in a separate document titled ‘NHANES - NCHS Research Ethics Review Board Approval’. Informed consent was obtained from all subjects and/or their legal guardians.

**Figure 1:**
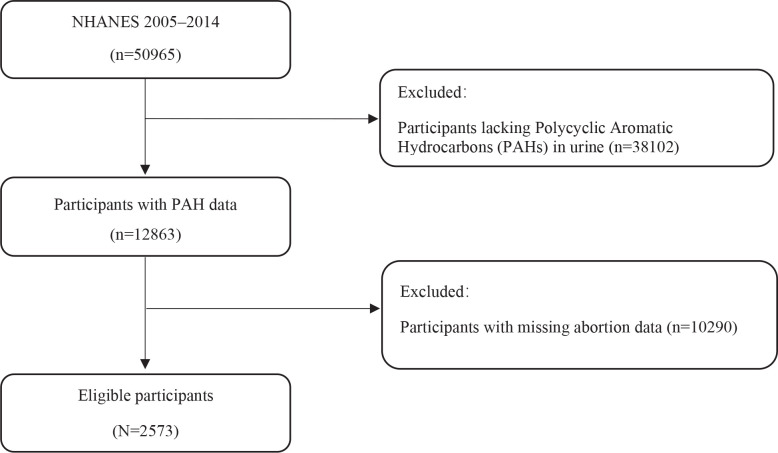
Baseline characteristics of women in the NHANES 2005–2014 cohort, stratified by miscarriage status (occurrence vs non-occurrence)

As shown in Figure 1, a total of 2573 participants were included in our study. Missing values for PAHs, number of pregnancies, and number of live births were excluded. By applying the WTINT2YR and WTMEC2YR weights, we calculated that this sample represents over 97 million people across the United States.

### Evaluation of PAHs: Main exposure factors

A previous study reported detailed information on the collection of PAHs in urine^16^. In brief, PAHs are easily metabolized after they enter the body and are present as major metabolites. This study involved the measurement of urinary metabolites, hydroxylated metabolites and glucosinolate metabolites. Urine samples for the measurement of urinary PAH metabolites were collected by specialized staff at a mobile testing center. Metabolite levels were determined using GC–HRMS. The details on ‘lower level of detection and relevant metrics of accuracy for measurement of PAHs’ are specified in the uploaded file Laboratory Procedure Manual-PAHs (Supplementary file). The following PAHs were included in our study: 1-hydroxynaphthalene, 2-hydroxynaphthalene, 3-hydroxyfluorene, 2-hydroxyfluorene, 1-hydroxyphenanthrene and 1-hydroxypyrene.

### Definition of miscarriage

Miscarriage is a common obstetrical event generally defined as the loss of a pregnancy before the fetus reaches viability^11^. The sample included in this study included individuals who responded to questions RHQ160 and RHQ171 in the questionnaire. In this study, miscarriage was defined as a number of pregnancies greater than the number of live births. When miscarriage was considered a continuous variable, it was expressed as the number of miscarriages. The categorical variable was expressed as the absence of miscarriage or miscarriage. The study included a broader range of pregnancy loss cases than strictly defined spontaneous miscarriage, such as induced abortions. However, by incorporating an unselected sample from the general population, the analysis better reflects the full spectrum of pregnancy loss experiences in society, enhancing the representativeness of our findings.

### Assessment of covariates

Demographic data, including age, race, education level, and the household poverty-to-income ratio (PIR), were obtained from the NHANES demographic questionnaire. Age and the PIR are continuous variables, and the others are categorical variables. Race was categorized as Mexican American, Non-Hispanic Black, Non-Hispanic White, Other Hispanic or Other Race. The PIR is calculated as the total family income divided by the poverty threshold for the interview year. Low income was defined as PIR≤1.300, medium income was defined as PIR=1.301–3.500, and high income was defined as PIR≥3.501. Education level was classified as lower than high school, completed high school, and higher than high school. In addition, other risk factors, such as hypertension, hyperlipidemia, cardiovascular disease (CVD), chronic kidney disease (CKD), BMI, smoking status and alcohol consumption, were included (Supplementary file Appendix 1).

### Statistical analysis

In the baseline characteristics table, the Mann-Whitney U test or one-way ANOVA was used for continuous variables, and the chi-square test was used for categorical variables. Frequencies and percentages were used to present categorical variables, while means (standard errors) were used to present continuous variables. Logistic regression models were used to analyze the ratio of PAHs to miscarriages (ORs) and 95% confidence intervals (CIs). PAH metabolite levels were divided into four quartiles (with the 25th quartile as the reference), and four models were developed with the aim of determining whether there was a dose-response relationship between exposure to PAHs and miscarriage and whether it was affected by confounding factors. The specific concentrations of the PAH quartiles are shown in Supplementary file Appendix 2. The crude model was unadjusted for any variable. Model 1: adjusted for age and ethnicity. Model 2: adjusted as in Model 1 plus education level, CKD status, and CVD incidence. Model 3: adjusted as in Model 2 plus alcohol consumption, BMI, hyperlipidemia, and hypertension.

Before the RCS, because of the large range of values and skewed distribution of PAH concentrations, log10 conversions were performed. The RCS was used to model and predict the relationship between the continuous variable PAH concentration and the dependent variable miscarriage. Within each segment, a linear regression model was employed to fit the curve, allowing for a visual understanding of the direction of the relationship between the two variables. In our study, RCS was implemented to further explore the dose-response relationship between PAHs and miscarriage and whether there was a nonlinear relationship. We used 3 knots in the RCS analysis. The p-values for the nonlinear trends were calculated using the Wald test on the RCS coefficients. To verify the stability of the results, stratified analyses were also implemented. In this study, statistical analysis was generated from R version 4.3.2, and p<0.05 was considered as the significance criterion. All tests were two-tailed.

## RESULTS

### Demographic characteristics of participants

Table 1 shows the baseline characteristics of participants according to miscarriage status in the NHANES 2005–2014. The mean age of the participants was 50.36 years. In the miscarriage population, the mean age was 49.39 years, which was younger than that in the absence of miscarriage population (p=0.02). The PIR was 2.68, which was relatively low (p=0.001), implying that a lower income contributed to the risk of miscarriage; the percentage of those who were still currently smoking was 24.40%, which was much greater than that of the absence of miscarriage population (18.75%). In contrast, there were no significant differences regarding some underlying diseases, such as CKD, CVD, hyperlipidemia, or hypertension (p*>*0.05). BMI and education level were also not significantly associated with miscarriage (p*>*0.05).

**Table 1:** Baseline characteristics of women in the NHANES 2005–2014 cohort, stratified by miscarriage status (N=2573)

*Characteristics*	*Total*	*Miscarriage*		*p*
		*No*	*Yes*	
**Age** (years)	50.36 (49.52–51.20)	51.13 (49.92–52.35)	49.39 (48.38–50.40)	0.02
**BMI** (kg/m^2^)	28.92 (28.58–29.25)	28.98 (28.51–29.45)	28.84 (28.28–29.40)	0.73
**PIR**	2.84 (2.75–2.94)	2.97 (2.86–3.08)	2.68 (2.54–2.82)	0.001
**Miscarriage**	0.78 (0.70–0.86)	0.00 (0.00–0.00)	1.75 (1.61–1.90)	<0.0001
**PAH**				
1-Hydroxynaphthalene (ng/L)	79895.07 (27134.81–132655.32)	103336.77 (14378.42–192295.13)	50596.91 (3452.76–97741.05)	0.31
2-Hydroxynaphthalene (ng/L)	7848.97 (7212.45–8485.49)	7183.17 (6462.75–7903.59)	8681.31 (7784.41–9578.21)	0.004
3-Hydroxyfluorene (ng/L)	286.98 (252.59–321.38)	260.82 (218.24–303.40)	320.07 (273.55–366.59)	0.04
2-Hydroxyfluorene (ng/L)	582.25 (515.05–649.45)	535.62 (459.96–611.28)	640.68 (541.79–739.57)	0.06
1-Hydroxyphenanthrene (ng/L)	195.45 (181.37–209.53)	188.84 (170.55–207.12)	203.72 (183.93–223.50)	0.26
1-Hydroxypyrene (ng/L)	189.03 (168.77–209.28)	172.94 (153.42–192.47)	209.22 (173.26–245.17)	0.07
**Race**				<0.001
Mexican American	8.81 (6.87–10.76)	9.54 (7.05–12.02)	7.91 (5.84–9.99)	
Non-Hispanic Black	12.16 (10.30–14.02)	10.03 (7.91–12.15)	14.83 (12.10–17.56)	
Non-Hispanic White	67.91 (60.66–75.16)	70.77 (66.97–74.57)	64.33 (59.58–69.08)	
Other Hispanic	4.76 (3.61– 5.91)	4.33 (2.88–5.78)	5.29 (3.96–6.62)	
Other Race	6.36 (4.91– 7.80)	5.34 (3.81–6.86)	7.63 (5.33–9.94)	
**Education level**				0.7
High school or lower	19.49 (17.43–21.55)	19.38 (17.14–21.63)	19.62 (16.99–22.26)	
Completed high school	25.72 (22.60–28.84)	26.66 (24.18–29.13)	24.54 (21.23–27.86)	
High school or higher	54.64 (50.29–59.00)	53.79 (50.65–56.94)	55.71 (51.68–59.74)	
**Smoking status**				0.01
Former	21.71 (19.55–23.86)	22.03 (19.80–24.26)	21.39 (18.76–24.03)	
Never	56.89 (52.71–61.06)	59.22 (56.44–62.01)	54.20 (51.12–57.28)	
Current	21.22 (18.64–23.81)	18.75 (15.62–21.88)	24.40 (21.79–27.02)	
**Alcohol**				0.11
Former	17.43 (15.40–19.47)	18.04 (15.15–20.93)	16.79 (14.23–19.36)	
Heavy	15.90 (13.63–18.18)	14.76 (12.13–17.39)	17.44 (14.58–20.30)	
Mild	31.35 (28.01–34.68)	33.02 (29.41–36.63)	29.48 (26.46–32.49)	
Moderate	19.37 (16.82–21.91)	17.56 (14.81–20.30)	21.77 (18.67–24.87)	
Never	15.64 (13.84–17.44)	16.62 (14.58–18.67)	14.51 (11.52–17.51)	
**CVD**				0.81
No	91.12 (85.20–97.05)	91.44 (89.65–93.23)	91.09 (89.13–93.04)	
Yes	8.70 (7.35–10.06)	8.56 (6.77–10.35)	8.91 (6.96–10.87)	
**CKD**				0.8
No	80.46 (74.97–85.94)	82.60 (80.30–84.90)	83.08 (80.53–85.63)	
Yes	16.70 (14.90–18.50)	17.40 (15.10–19.70)	16.92 (14.37–19.47)	
**Hyperlipidemia**				0.2
No	28.61 (25.65–31.58)	27.20 (24.21–30.20)	30.38 (26.75–34.01)	
Yes	71.39 (66.40–76.37)	72.80 (69.80–75.79)	69.62 (65.99–73.25)	
**Hypertension**				0.08
No	59.26 (54.69–63.83)	57.53 (54.19–60.86)	61.42 (58.01–64.83)	
Yes	40.74 (37.05–44.44)	42.47 (39.14–45.81)	38.58 (35.17–41.99)	

Data are expressed as mean (95% CI) for continuous variables and percent (95% CI) for categorical variables. BMI: body mass index. PIR: poverty income ratio. CVD: cardiovascular diseases. CKD: chronic kidney disease. For continuous variables, both the independent samples t-test and the Mann-Whitney U test were used. For categorical variables, the chi-squared test was applied.

### Relationships between PAHs and miscarriage rates analyzed using various statistical models

As shown in Table 2, we performed univariable logistic regression analysis between PAHs and miscarriage risk and divided the results into crude models and three models adjusting for different variables. Using the lowest 25th percentile (Q1) as a reference, the ORs and CIs for the highest quartile (Q4) of the following substances were 1-hydroxynaphthalene (OR=1.14; 95% CI: 0.86–1.51, p=0.35), 2-hydroxynaphthalene (OR=1.45; 95% CI: 1.16–1.81, p=0.002), 3-hydroxyfluorene (OR=1.36; 95% CI: 1.05–1.77, p=0.02), 2-hydroxyfluorene (OR=1.34; 95% CI: 1.04–1.73, p=0.002), 1-hydroxyphenanthrene (OR=1.32; 95% CI: 1.00–1.74, p=0.05), and 1-hydroxypyrene (OR=1.47; 95% CI: 1.16–1.86, p=0.002). However, at lower concentrations (Q1–Q3), PAHs did not affect miscarriage (p>0.05). This indicates that, in addition to 1-hydroxynaphthalene, the remaining PAHs examined in this study were found to have a risk impact on the occurrence of miscarriage. With increasing concentrations in the body, PAHs promote miscarriage.

**Table 2:** Univariable logistic regression analysis of miscarriage risk factors in NHANES 2005–2014 (N=2573)

*Variable*	*Estimate*	*p*	*OR*	*95% CI*
**Age** (years)	-0.01	0.03	0.99	0.99 (0.99–1.00)
**BMI** (kg/m^2^)	0	0.73	1	1.00 (0.98–1.01)
**PIR**	-0.11	0.001	0.9	0.90 (0.84–0.96)
**Race**				
Mexican American ®				
Non-Hispanic Black	0.58	<0.001	1.78	1.78 (1.31–2.43)
Non-Hispanic White	0.09	0.49	1.1	1.10 (0.84–1.43)
Other Hispanic	0.39	0.05	1.47	1.47 (0.99–2.18)
Other Race	0.54	0.03	1.72	1.72 (1.05–2.82)
**Education level**				
High school or lower ®				
Completed high school	-0.09	0.42	0.91	0.91 (0.72–1.15)
High school or higher	0.02	0.82	1.02	1.02 (0.84–1.25)
**Smoking status**				
Former ®				
Never	-0.06	0.57	0.94	0.94 (0.77–1.16)
Current	0.29	0.05	1.34	1.34 (1.00–1.80)
**Alcohol**				
Former ®				
Heavy	0.24	0.22	1.27	1.27 (0.87–1.86)
Mild	-0.04	0.81	0.96	0.96 (0.68–1.35)
Moderate	0.29	0.12	1.33	1.33 (0.92–1.92)
Never	-0.06	0.71	0.94	0.94 (0.66–1.33)
**CVD**				
No ®				
Yes	-0.03	0.80	0.97	0.97 (0.74–1.26)
**CKD**				
No ®				
Yes	0.04	0.81	1.04	1.04 (0.73–1.49)
**Hyperlipidemia**				
No ®				
Yes	-0.16	0.20	0.86	0.86 (0.68–1.09)
**Hypertension**				
No ®				
Yes	-0.16	0.08	0.85	0.85 (0.71–1.02)
**1-Hydroxynaphthalene**				
Q1 ®				
Q2	-0.06	0.67	0.94	0.94 (0.72–1.24)
Q3	0.02	0.89	1.02	1.02 (0.77–1.36)
Q4	0.13	0.35	1.14	1.14 (0.86–1.51)
**2-Hydroxynaphthalene**				
Q1 ®				
Q2	0.32	0.06	1.37	1.37 (0.99–1.91)
Q3	0.18	0.29	1.2	1.20 (0.85–1.68)
Q4	0.37	0.002	1.45	1.45 (1.16–1.81)
**3-Hydroxyfluorene**				
Q1 ®				
Q2	-0.02	0.89	0.98	0.98 (0.71–1.34)
Q3	0.11	0.49	1.11	1.11 (0.82–1.52)
Q4	0.31	0.02	1.36	1.36 (1.05–1.77)
**2-Hydroxyfluorene**				
Q1 ®				
Q2	0.09	0.54	1.09	1.09 (0.83–1.44)
Q3	0.07	0.68	1.07	1.07 (0.77–1.48)
Q4	0.29	0.02	1.34	1.34 (1.04–1.73)
**1-Hydroxyphenanthrene**				
Q1 ®				
Q2	0.07	0.59	1.07	1.07 (0.83–1.38)
Q3	0.13	0.34	1.14	1.14 (0.87–1.50)
Q4	0.28	0.05	1.32	1.32 (1.00–1.74)
**1-Hydroxypyrene**				
Q1 ®				
Q2	0.16	0.28	1.17	1.17 (0.88–1.55)
Q3	0.15	0.26	1.17	1.17 (0.89–1.53)
Q4	0.39	0.002	1.47	1.47 (1.16–1.86)

BMI: body mass index. PIR: poverty income ratio. CVD: cardiovascular diseases. CKD: chronic kidney disease. Q1–Q4: quartiles. ® Reference categories.

Then, we adjusted the three models for different confounders, eliminated their potential effects on the outcome variables, and performed multivariable logistic regressions to further observe the robustness of the findings (Table 3). Of all the metabolites of PAHs analyzed, only 2-hydroxynaphthalene consistently emerged as a risk factor for miscarriage in all three models tested, namely, Model 1 (adjusted for age and ethnicity): (AOR=1.33; 95% CI: 1.06–1.67, p=0.02), Model 2 (adjusted for age, ethnicity, education level, CKD, and CVD) (AOR=1.30; 95% CI: 1.03–1.64, p=0.03) , and Model 3 (adjusted for age, ethnicity, education, CKD, CVD, BMI, alcohol consumption, hyperlipidemia, and hypertension) (AOR=1.31; 95% CI: 1.02–1.69, p=0.04). Therefore, it can be inferred that 2-hydroxynaphthalene posed a constant risk for miscarriage, regardless of the occurrence of any confounding factors.

**Table 3:** Multivariable logistic regression analysis of miscarriage risk associated with quartile concentrations of polycyclic aromatic hydrocarbons (PAHs) in NHANES 2005–2014 (N=2573)

*Variable*	*Model 1*			*Model 2*			*Model 3*		
	*p*	*AOR*	*95% CI*	*p*	*AOR*	*95% CI*	*p*	*AOR*	*95 % CI*
**2-Hydroxynaphthalene**									
Q1 ®		1			1			1	
Q2	>0.05	1.33	1.325 (0.950–1.850)	>0.05	1.33	1.327 (0.937–1.879)	>0.05	1.35	1.346 (0.947–1.912)
Q3	>0.05	1.12	1.117 (0.793–1.575)	>0.05	1.12	1.123 (0.784–1.608)	>0.05	1.11	1.114 (0.772–1.608)
Q4	0.02	1.33	1.332 (1.060–1.674)	0.03	1.30	1.300 (1.028–1.645)	0.04	1.31	1.313 (1.018–1.694)
**1-Hydroxynaphthalene**									
Q1 ®		1			1			1	
Q2	>0.05	0.92	0.921 (0.698–1.216)	>0.05	0.94	0.943 (0.716–1.242)	>0.05	0.94	0.944 (0.718–1.239)
Q3	>0.05	0.99	0.994 (0.743–1.329)	>0.05	1.03	1.030 (0.759–1.397)	>0.05	1.04	1.038 (0.757–1.423)
Q4	>0.05	1.08	1.080 (0.813–1.435)	>0.05	1.11	1.108 (0.826–1.488)	>0.05	1.10	1.097 (0.803–1.499)
**3-Hydroxyfluorene**									
Q1 ®		1			1			1	
Q2	>0.05	0.96	0.961 (0.700–1.318)	>0.05	0.94	0.944 (0.669–1.330)	>0.05	0.97	0.969 (0.684–1.373)
Q3	>0.05	1.05	1.051 (0.763–1.446)	>0.05	1.04	1.038 (0.740–1.455)	>0.05	1.06	1.055 (0.745–1.493)
Q4	>0.05	1.26	1.258 (0.966–1.637)	>0.05	1.25	1.253 (0.947–1.658)	>0.05	1.26	1.262 (0.937–1.702)
**2-Hydroxyfluorene**									
Q1 ®		1			1			1	
Q2	>0.05	1.07	1.066 (0.806–1.408)	>0.05	1.09	1.093 (0.816–1.465)	>0.05	1.12	1.115 (0.826–1.505)
Q3	>0.05	1.01	1.010 (0.726–1.405)	>0.05	1.03	1.026 (0.726–1.451)	>0.05	1.06	1.055 (0.737–1.510)
Q4	>0.05	1.23	1.229 (0.956–1.579)	>0.05	1.25	1.249 (0.958–1.629)	>0.05	1.26	1.260 (0.960–1.653)
**1-Hydroxyphenanthrene**									
Q1 ®		1			1			1	
Q2	>0.05	1.04	1.040 (0.805–1.344)	>0.05	1.06	1.063 (0.810–1.394)	>0.05	1.08	1.082 (0.815–1.438)
Q3	>0.05	1.10	1.095 (0.827–1.448)	>0.05	1.09	1.093 (0.809–1.477)	>0.05	1.10	1.097 (0.815–1.476)
Q4	>0.05	1.25	1.246 (0.941–1.651)	>0.05	1.27	1.269 (0.937–1.720)	>0.05	1.29	1.289 (0.941–1.767)
**1-Hydroxypyrene**									
Q1 ®		1			1			1	
Q2	>0.05	1.14	1.143 (0.862–1.516)	>0.05	1.15	1.148 (0.852–1.548)	>0.05	1.15	1.149 (0.844–1.565)
Q3	>0.05	1.09	1.091 (0.825–1.442)	>0.05	1.09	1.091 (0.819–1.454)	>0.05	1.09	1.088 (0.802–1.475)
Q4	0.03	1.31	1.310 (1.024–1.676)	>0.05	1.27	1.273 (0.985–1.645)	>0.05	1.26	1.262 (0.963–1.654)

AOR: adjusted odds ratio. Model 1: adjusted for age and ethnicity. Model 2: adjusted as for Model 1 plus education, CKD status and CVD incidence. Model 3: adjusted as for Model 2 plus alcohol consumption, BMI, hyperlipidemia, and hypertension. Q1–Q4: quartiles. ® Reference categories.

We also used the concentration of PAHs and the number of miscarriages as continuous variables to perform linear regressions (Table 4). There was a linear relationship between 2-hydroxynaphthalene (t=2.67, p=0.01), 3-hydroxyfluorene (t=2.67, p=0.01), 2-hydroxyfluorene (t=2.82, p=0.01), 1-hydroxyphenanthrene (t=2.40, p=0.02), 1-hydroxypyrene (t=3.63, p<0.001) and miscarriage, while 1-hydroxynaphthalene was not related (p=0.19).

**Table 4:** Linear regression analysis of the dose-response relationship between PAH concentrations and miscarriage in NHANES 2005–2014 (N=2573)

*PAH*	*Estimate*	*SE*	*t*	*p (>|t|)*	*95 % CI*
1-Hydroxynaphthalene	0.02	0.01	1.34	0.19	0.0179 (-0.0088–0.0446)
2-Hydroxynaphthalene	0.07	0.03	2.67	0.01	0.0666 (0.0166–0.1165)
3-Hydroxyfluorene	0.07	0.03	2.67	0.01	0.0696 (0.0174–0.1218)
2-Hydroxyfluorene	0.08	0.03	2.82	0.01	0.0775 (0.0225–0.1325)
1-Hydroxyphenanthrene	0.07	0.03	2.40	0.02	0.0716 (0.0118–0.1313)
1-Hydroxypyrene	0.08	0.02	3.63	<0.001	0.0808 (0.0363–0.1253)

SE: standard error.

**Figure 2:**
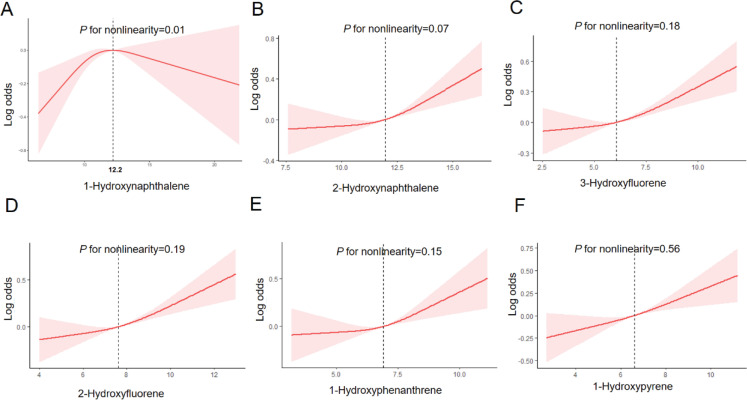
Restricted cubic spline (RCS) analysis of the dose-response relationship between log_2_-transformed polycyclic aromatic hydrocarbon (PAH) concentrations and miscarriage risk in NHANES 2005–2014: A) 1-Hydroxynaphthalene, B) 2-Hydroxynaphthalene, C) 3-Hydroxyfluorene, D) 2-Hydroxyfluorene, E) 1-Hydroxyphenanthrene, and F) 1-Hydroxypyrene

Then, to further clarify the specific directional relationship between PAHs and miscarriage and whether there was a nonlinear relationship, the RCS was implemented. 1-hydroxynaphthalene (p for nonlinearity=0.01) and miscarriage had a clear nonlinear relationship and presented an ‘n’ shape. The cutoff value for log2 (1-hydroxynaphthalene) was 12.20 ng/L (Figure 2A). It was concluded that the risk of miscarriage increased with increasing concentrations of 1-hydroxynaphthalene below 4705 ng/L *in vivo*. Above 4705 ng/L, the risk of miscarriage decreases with increasing concentrations in the body. The other PAHs exhibited a monotonically increasing linear relationship (p for nonlinear >0.05), which was consistent with the previous linear regression results (Figures 2B–2F). In conclusion, there was a nonlinear relationship between 1-hydroxynaphthalene and miscarriage, with higher concentrations leading to a greater risk of miscarriage before the cutoff value and higher concentrations leading to a lower risk after the cutoff value. Moreover, 2-hydroxynaphthalene, 3-hydroxyfluorene, 3-hydroxyfluorene, 2-hydroxyfluorene, 1-hydroxyphenanthrene and 1-hydroxypyrene exhibited a clear dose-dependent relationship, i.e. as the concentration of these endocrine disruptors increased, the risk of miscarriage increased.

### Stratified analysis

Further stratified analyses were performed by race, smoking status, alcohol consumption, CVD, and CKD (Supplementary file Appendix 3). The results showed that BMI, smoking status, and age did not interact with miscarriage risk after adjusting for confounding variables (p for interaction >0.05), apart from the interaction between smoking and miscarriage when analyzed at the race level (p=0.02). These findings demonstrated that reducing exposure to smoke may alleviate the risk of miscarriage. These findings further demonstrated the robustness of the results and that the relationship between 2-hydroxynaphthalene and miscarriage was not subject to interactions.

## DISCUSSION

Our findings suggest a positive association between certain PAHs and miscarriage. Urinary concentrations of 2-hydroxynaphthalene, 3-hydroxyfluorene, 2-hydroxyfluorene, 1-hydroxyphenanthrene, and 1-hydroxypyrene were positively linearly correlated with the number of miscarriages, and these concentrations were risk factors for miscarriage at the highest quartile level. The results for 2-hydroxynaphthalene were more robust than those for the other tested analytes and not affected by confounders. In addition, we performed statistical methods such as RCS and stratified analyses. 1-hydroxynaphthalene has an n-shaped relationship with miscarriage. Notably, a recent NHANES analysis documented an inverted U-shaped (n-shaped) association between 1-hydroxyphenanthrene and hyperlipidemia, with risk increasing at low doses and decreasing at high doses – paralleling our findings for 1-hydroxynaphthalene and miscarriage^17^. This consistency across different PAH metabolites and health endpoints suggests that nonlinear dose responses may be a general feature of PAH toxicity, warranting further investigation into shared biological pathways, such as aryl hydrocarbon receptor (AhR)-mediated oxidative stress or metabolic enzyme saturation effects. This is one of the first studies to examine the relationship between PAH exposure levels and the risk of miscarriage and may inform the exploration of risk factors for miscarriage.

PAHs can be used daily and can affect different systems of the body. Several studies have explored the association of PAHs with mothers and children, the reproductive system, asthma, lung cancer, breast cancer, and other diseases, and some interesting results have been found. Among the effects on mothers and children, PAHs can be found in the placenta as well as in breast milk and can adversely affect infants^18,19^. In studies of female reproductive disorders, PAHs have been shown to exacerbate reproductive toxicity, causing infertility, polycystic ovary syndrome, and premature ovarian failure^20^. Additionally, a cohort study conducted among college students in Chongqing, China, explored associations between PAH exposure and male reproductive parameters, specifically normal sperm morphology, sperm viability, and the proportion of spermatozoa with high DNA/chromosomal content^21^. Among the studies on asthma, there are retrospective studies on children: PAH exposure increases the prevalence of asthma in children, and asthma is the most common chronic disease in children living in developed countries^22^. A specific PAH product, 2-hydroxyphenanthrene, is associated with female asthma when it is doubled^23^. In addition, PAHs are also associated with a variety of cancers, such as lung and trachea cancers. PAH exposure may further promote lung carcinogenesis by producing epigenetic changes^24^. Among the effects on breast cancer, in overweight girls, PAHs affect the timing of pubertal development, which is an important risk factor for breast cancer^25,26^, and long-term exposure to PAHs may increase the risk of breast cancer, especially for women with a family history of breast cancer^27,28^. In conclusion, PAHs have more negative impacts on multiple body systems, both in animals and humans and in men and women. However, studies on the effects of PAHs on the reproductive system have not been complete, and the specific effects of PAHs on miscarriage have not been found.

The association between PAH and miscarriage may be partially mediated by smoking, as tobacco smoke contains high levels of carcinogenic PAHs^29^. Specifically, studies on female reproductive disorders have confirmed that PAHs exacerbate reproductive toxicity^20^. For example, benzo(a)pyrene (a type of PAH) disrupts folliculogenesis and ovulation in rats, leading to adverse reproductive outcomes^30^. The mechanism may be related to the aryl hydrocarbon receptor (AhR) pathway – AhR is a transcription factor expressed in ovarian tissues, and PAHs inhibit its regulation of follicular growth, stimulating follicular apoptosis and increasing the risk of polycystic ovary syndrome (POF)^31^ . Clinically, cohort studies have shown that POF significantly elevates the risk of early spontaneous miscarriage and pregnancy loss, while PAHs further exacerbate this pathological process by interfering with key reproductive hormones such as luteinizing hormone and follicle-stimulating hormone^32^. More specific scientific mechanisms require integration of clinical and basic research to validate.

In addition to PAHs, in our univariable logistic regression analysis, age and poverty index were also found to influence miscarriage prevalence; using Mexican Americans as a reference, non-Hispanic black individuals exhibited a significantly higher likelihood of miscarriage. Smoking is also a risk factor for the development of miscarriage. One contributing factor is attributed to the complex mixture of chemicals in tobacco smoke, which can lead to alterations in fertility and an accelerated onset of menopause^33,34^. In addition, the present study focused only on the possible effects of pregnancy outcomes in women, first, because of the lack of information on the criteria for judging male infertility, and second, because women are more likely to be affected by the toxicity of PAHs through the CYP1A1 enzyme, the ability to repair DNA damage is weaker in women than in men^35^.

This was a population-based cross-sectional study, and we had a large sample size of 2573 US women who participated in NHANES in the period 2005–2014. We performed multiple linear regressions with PAH concentration and number of miscarriages as continuous variables and logistic regression with PAH quartiles and miscarriages as dichotomous endpoints. RCS was used to analyze whether there was a nonlinear relationship and to determine whether there was a correlation between the two variables. This is the first study to examine PAHs and miscarriage using a large sample population.

### Limitations

This study has several limitations. First, because this was a cross-sectional study, we cannot explore the exact causal relationship, and additional clinical trials and basic experiments are needed to determine the exact mechanism involved. Second, although we adjusted for potential confounders, residual confounding may persist due to unmeasured factors such as specific occupational exposures. Third, measurement error in PAHs could affect results. Moreover, the definition of miscarriage adopted in this study was not rigorous. It defined miscarriage as the difference between the number of reported pregnancies and live births. This broad definition potentially includes ectopic pregnancies, pregnancy terminations, stillbirths, and artificial miscarriages due to subjective factors, none of which were distinguished or accounted for in this study. The mean age of the women included in the analysis is 50 years, which may introduce recall bias regarding their reproductive period. Finally, the study population was restricted to the US, and geographical, cultural, and legal disparities may limit generalizability to other regions. In conclusion, this large-scale cross-sectional study is one of the first to propose that high concentrations of tobacco-derived PAHs are risk factors for miscarriage and provides guidance for exploring the causes of miscarriage, particularly through their association with tobacco smoke exposure.

## CONCLUSIONS

Our study of 2573 participants from NHANES 2005–2014 revealed that certain PAHs, like 2-hydroxynaphthalene, 3-hydroxyfluorene, 2-hydroxyfluorene, 1-hydroxyphenanthrene, and 1-hydroxypyrene, increase miscarriage risk as their concentrations rise in the body. 2-hydroxynaphthalene was a consistent risk factor across adjusted models. There was a ‘n-shaped’ relationship between 1-hydroxynaphthalene and miscarriage, with other PAHs showing a linear dose- dependent increase in risk. Stratified analysis confirmed result robustness. In summary, this finding contributes to public health interventions targeting reduction of tobacco-derived PAH exposure among women of reproductive age, such as enforcing stricter regulations on tobacco smoke-related emissions, promoting smoke free environments, while advocating for targeted health education to enhance awareness of tobacco-induced PAH risks.

## Supplementary Material



## Data Availability

The data supporting this research can be found in the Supplementary file and in the NHANES database, https://www.cdc.gov/nchs/nhanes/, accessed on 17 March 2023.
