# Supplementary material for: Polycyclic aromatic hydrocarbons exposure as a potential risk factor for miscarriage among women in the United States: A secondary dataset analysis of NHANES data for the period 2005–2014
Source: Tob Induc Dis. 2025 Aug 8;23:10.18332/tid/205903. doi: 10.18332/tid/205903 (PMC12332853; doi:10.18332/tid/205903)
Supplement: Supplementary file 1 [file TID-23-115-s1.pdf]

Laboratory Procedure Manual

Analyte: **Eight monohydroxy-polycyclic aromatic hydrocarbons: 1-hydroxynaphthalene, 2-hydroxynaphthalene, 2-hydroxyfluorene, 3-hydroxyfluorene, 1-hydroxyphenanthrene, 2- & 3-hydroxyphenanthrene, 1-hydroxypyrene**

Matrix: **Urine**

Method: **Isotope Dilution Online Solid Phase Extraction High Performance Liquid Chromatography/Tandem Mass Spectrometry (online SPE-HPLC-MS/MS)**

Method No: **6705.02**

As performed by:

Organic Analytical Toxicology Branch
Division of Laboratory Sciences

National Center for Environmental Health

Contact:

Xiaoyun (Sherry) Ye, M.S.
Phone: 770.488.7502
Email: XYe@cdc.gov

James L. Pirkle, M.D., Ph.D.
Director, Division of Laboratory Sciences

Important Information for Users

The Centers for Disease Control and Prevention (CDC) periodically refines these laboratory methods. It is the responsibility of the user to contact the person listed on the title page of each write-up before using the analytical method to find out whether any changes have been made and what revisions, if any, have been incorporated.

Public Release Data Set Information

This document details the Lab Protocol for testing the items listed in the following table:

Data File Name	Variable Name	SAS Label
PAH_H and PAHS_H	URXPO1	1-napthol (ng/L)
	URXPO2	2-napthol (ng/L)
	URXPO3	3-hydroxyfluorene (ng/L)
	URXPO4	2-hydroxyfluorene (ng/L)
	URXPO6	1-hydroxyphenanthrene (ng/L)
	URXP10	1-hydroxypyrene (ng/L)
	URXP25	2-hydroxyphenanthrene and 3-hydroxyphenanthrene (ng/L)

1. Clinical Relevance and Summary of Test Principle

a. Clinical Relevance

Polycyclic aromatic hydrocarbons (PAHs) are a class of ubiquitous environmental contaminants formed during incomplete combustion processes. Many of them have been identified as suspected human carcinogens (1), but threshold levels for carcinogenicity have not been determined for most PAHs. Occupational exposure may occur through work involving diesel fuels and coal tars such as paving and roofing. Possible environmental exposures include smoking, diet, smog and forest fires (2). Because of potential widespread human exposure and potential risk to health, biomonitoring of PAHs is relevant for environmental public health (3,4). Upon exposure, PAHs are metabolized in humans; some of these metabolites are excreted in urine. Information on the concentration of metabolites of PAHs in people is important for understanding human exposure.

b. Test Principle

The test principle utilizes high performance liquid chromatography-electrospray ionization-tandem mass spectrometry (HPLC-ESI-MS/MS) for the quantitative detection of several monohydroxylated metabolites of PAHs (OH-PAHs) in urine. The procedure involves enzymatic hydrolysis of glucuronidated/sulfated OH-PAH metabolites, centrifugation, dilution and analysis using isotope dilution online solid phase extraction (SPE) coupled with HPLC-ESI-MS/MS. Ion transitions specific to each analyte and carbon-13 labeled internal standards are monitored, and the abundances of each ion are measured. The analytes measured in this procedure are shown in Table 1.

Table 1. Analytes, their parent compounds, and their abbreviations.

No.	Metabolite/Analyte	Parent PAH	Abbreviation	Note
1	1-hydroxynaphthalene	Naphthalene	1-NAP	
2	2-hydroxynaphthalene		2-NAP	
3	2-hydroxyfluorene	Fluorene	2-FLU	
4	3-hydroxyfluorene		3-FLU	
5	1-hydroxyphenanthrene	Phenanthrene	1-PHE	2-, 3-PHE measured together
6	2-hydroxyphenanthrene		2-PHE	
7	3-hydroxyphenanthrene		3-PHE	
8	1-hydroxypyrene	Pyrene	1-PYR	

2. Safety Precautions

a. Reagent Toxicity or Carcinogenicity

Some of the reagents needed to perform this procedure are toxic. Special care must be taken to avoid inhalation or dermal exposure to these reagents.

β-Glucuronidase is a known sensitizer. Prolonged or repeated exposure to the sensitizer may cause allergic reactions in certain sensitive individuals.

Note: Material Safety Data Sheets (MSDS) for the chemicals and solvents used in this procedure can be found at http://intranet.cdc.gov/CHATS/OHS/chem_search.asp or http://www.msdsxchange.com/english/index.cfm. Laboratory personnel are advised to review the MSDS before using chemicals and solvents.

b. Radioactive Hazards

There are no radioactive hazards associated with this procedure.

c. Microbiological Hazards

Although urine is generally regarded as less infectious than serum, the possibility of being exposed to various microbiological hazards exists. Appropriate measures must be taken to avoid any direct contact with the specimen. CDC recommends a Hepatitis B vaccination series and a baseline serum test for health care and laboratory workers who might be exposed to human fluids and tissues. Laboratory workers observe universal precautions to prevent any direct contact with the specimen. Also, laboratory personnel handling human fluids and tissues are required to take the “Bloodborne Pathogens Training” course and subsequent refresher courses offered at CDC to insure proper compliance with CDC safe work place requirements.

d. Mechanical Hazards

There are only minimal mechanical hazards when performing this procedure using standard safety practices. Laboratory analysts must read and follow the manufacturers’ information regarding safe operation of equipment. Avoid direct contact with the mechanical and electronic components of the mass spectrometer unless all power to the instrument is off. Generally, mechanical and electronic maintenance and repair must only be performed by qualified technicians. Avoid contact with the heated surfaces of the mass spectrometer (e.g., interface). Multiple solvent bottles (1L) are located on and around the instruments. Numerous tubing lines are used to transfer solvents from storage bottles to the instrument and the waste bottles. Precautions must be used when working in these areas.

e. Protective Equipment

Standard safety precautions must be followed when performing this procedure, including the use of a lab coat/disposable gown, safety glasses, appropriate gloves, and chemical fume hood. Refer to the laboratory Chemical Hygiene Plan and CDC Division of Laboratory Sciences safety policies and procedures for details related to specific activities, reagents, or agents.

f. Training

Formal training is necessary in the use of the Staccato® automated sampler, Agilent 1200 HPLC, AB Sciex 5500/6500 MS-MS, and online SPE system. Users are

required to read the operation manuals and demonstrate safe techniques in performing the method. Laboratorians involved in sample preparation must be trained for all sample preparation equipment, chemical handling, and have basic chemistry laboratory skills.

g. Personal Hygiene

Follow Universal Precautions. Care must be taken when handling chemicals or any biological specimen. Routine use of gloves and proper hand washing must be practiced. Refer to the laboratory Chemical Hygiene Plan and CDC Division of Laboratory Sciences safety policies and procedures for details related to specific activities, reagents, or agents.

h. Disposal of Wastes

Waste materials must be disposed of in compliance with laboratory, local, state, and federal regulations. Solvents and reagents must always be disposed of in an appropriate container clearly marked for waste products and temporarily stored in a chemical fume hood. Disposable items that come in direct contact with the biological specimens are to be placed in a biohazard autoclave bag that must be kept in appropriate containers until sealed and autoclaved. Needles, pipette tips and disposable syringes must be discarded into sharps containers and autoclaved. Contaminated surfaces should be disinfected with a freshly prepared bleach solution (e.g., ~0.5% available chlorine, or a 100 mL/L dilution of commercial sodium hypochlorite solution containing 5% available chloride). Any non-disposable glassware or equipment that comes in contact with biological samples must be washed with bleach solution before reuse or disposal. Any other non-disposable glassware must be washed and recycled or disposed of in an appropriate manner. To insure proper compliance with CDC requirements, laboratory personnel are required to take annual hazardous waste disposal training courses.

3. Computerization; Data-System Management

a. Software and Knowledge Requirements

Spiking of samples and hydrolysis of conjugates normally take place on a Staccato® Automated System controlled by the Perkin Elmer iLink and Maestro softwares. The SPE-HPLC-MS/MS system uses an Agilent 1200 series HPLC pump and AB Sciex 5500/6500 MS/MS, controlled by AB Sciex Analyst™ software, and an iChrom Symbiosis™ online SPE system, controlled by Sparklink® software. Analyte chromatographic peaks are integrated by MultiQuant™ or Analyst®. Results can be exported from Multiquant™ or Analyst®as text files which are subsequently processed using Excel, Access or SAS Enterprise Guide (SAS EG). Knowledge and experience with these software packages (or their equivalent) are required to utilize and maintain the data management structure.

b. Sample Information

Information pertaining to particular specimens is entered into the database (Access or STARLIMS) either manually or electronically using the files received from Sample

Logistics. The result file is transferred electronically into the database. No personal identifiers are used, only coded sample identifiers.

c. Data Maintenance

All sample and analytical data are reviewed for overall validity. The database is routinely backed up locally through the standard practices of the CDC network. The local area network manager can be contacted for emergency assistance.

4. Procedures for Collecting, Storing, and Handling Specimens; Criteria for Specimen Rejection

a. Special Instructions

No special instructions such as fasting or special diet are required.

b. Sample Collection

Urine specimens are collected from subjects in standard urine collection cups. Samples should be refrigerated as soon as possible, and preferably transferred to specimen vials within 24 hours of collection. If possible, a minimum of 2 milliliters of urine is collected and poured into vials (e.g., polypropylene, glass) with screw-cap tops. The specimens should be labeled, frozen at or below -20 ºC, and stored on dry ice for shipping. Special care must be taken to protect vials from breakage during shipment. At CDC, samples are kept frozen, preferably at -70 ºC, until and after analysis.

c. Sample Handling

Specimen handling conditions are outlined in the Division of Laboratory Sciences (DLS) protocol for urine collection and handling (e.g., copies available in branch, laboratory). In general, urine specimens should be transported and stored frozen. Once received, they should be frozen, preferably at -70 °C, until time for analysis. Portions of the sample that remain after analytical aliquots are withdrawn must be refrozen as soon as possible after use.

d. Sample Quantity

The regular sample size for analysis is 0.1 mL; the minimum amount of specimen generally required for a regular analysis is 0.05 mL.

e. Unacceptable Specimens

Specimens must be frozen when delivered to the lab. The minimum volume generally required for a single analysis is 0.1 mL. If either of these criteria is violated, the specimen may be rejected. Specimens can also be rejected if suspected of contamination due to improper collection procedures or devices. Specimen characteristics that may compromise test results include contamination of urine from improper handling. Samples with visible microbiological growth (e.g., mold, bacteria) might be inadequate for analysis. In case of rejected specimens, we

would request a second specimen if possible. A description of reasons for rejecting a sample must be recorded on the sample transfer sheet (e.g., low sample volume, leaking or damaged container).

5. Procedures for Microscopic Examinations; Criteria for Rejecting Inadequately Prepared Slides

Not applicable for this procedure.

6. Preparation of Reagents, Calibration Materials, Control Materials, and all Other Materials; Equipment and Instrumentation

a. Reagents and Sources

See Table 2.

Table 2. Reagents and suggested manufacturers

Reagent	Suggested Manufacturers*
Water (HPLC grade), acetonitrile, methanol	ThermoFisher Scientific, Inc., Waltham, MA
β–glucuronidase/arylsulfatase (H-1, powder enzyme), glacial acetic acid, sodium acetate, ascorbic acid, formic acid	Sigma-Aldrich Chemical, St. Louis, MO
$^{13}C_6$ 1-NAP, $^{13}C_6$ 2-NAP, $^{13}C_6$ 2-FLU, $^{13}C_6$ 3-FLU, $^{13}C_6$ 1-PHE, $^{13}C_6$ 2-PHE , $^{13}C_6$ 3-PHE, $^{13}C_6$ 1-PYR	Cambridge Isotope Laboratories, Andover, MA
1-NAP, 2-NAP, 1-PYR	Sigma-Aldrich Chemical, St. Louis, MO
2-FLU, 3-FLU, 1-PHE, 2-PHE, 3-PHE	Cambridge Isotope Laboratories, Andover, MA

* Products from other manufacturers with similar purity or specifications may be used.

b. Preparation of Reagents

1) Sodium Acetate Buffer Solution (~1 mol/L, pH 5.5±0.2)

Weigh sodium acetate and record in logbook. Transfer contents to a clean glass bottle and add necessary volume of de-ionized water (DI H_2O) to make a 1 mol/L solution. An example solution is 10.25 g sodium acetate diluted with 125 mL of DI water. Stir on a stir plate until the solid completely dissolves. Measure pH and record the value in logbook. Adjust the pH to 5.5 with glacial acetic acid; record final volume in logbook.

2) β-glucuronidase/arylsulfatase enzyme/Buffer solution (~10 g/L)

Weigh the needed amount of β-glucuronidase/arylsulfatase (H-1, powder enzyme) into a glass vial to have a final concentration of ~10 g/L. Add 1 mL of the sodium acetate buffer solution (1 mol/L, pH 5.5) for each 0.01 g of enzyme

and cap the vial (e.g. 0.40 g enzyme to 40 mL buffer). Place vial on a rotating mixer at ~40 rpm until the enzyme is completely dissolved. Store unused enzyme/buffer solution refrigerated for up to one week.

3) Ascorbic Acid Solution (~12.5 g/L)

Weigh L-ascorbic acid into a glass vial or test tube. Add 80 μL of deionized water for each milligram of ascorbic acid (e.g. 14 mg ascorbic acid to 11.2 mL water) and cap the vial. Place vial on a rotating mixer at 40 rpm until the solute is completely dissolved. Store unused ascorbic acid solution at room temperature for up to one week.

4) Synthetic Urine

For a 1L final volume, add the following chemicals in order, then fill to 1L with D.I. water. Store the solution in the refrigerator or freezer until use.

- 500 mL Water
- 3.8 g Potassium Chloride
- 8.5 g Sodium Chloride
- 24.5 g Urea
- 1.03 g Magnesium Sulfate ($MgSO_4.7H_2O$)
- 1.03 g Citric Acid
- 0.34 g Ascorbic Acid
- 1.18 g Potassium Phosphate, dibasic
- 1.4 g Creatinine
- 0.64 g Sodium Hydroxide (add slowly)
- 0.47 g Sodium Bicarbonate
- 0.28 mL Sulfuric Acid (concentrated.)

c. Preparation of Calibration Materials

Standard preparation is based on gravimetric and volumetric determination. Actual calculated concentrations based on weight are used in all data calculation and processing, and the actual preparation and final concentrations may slightly deviate from the normal procedure or target concentrations.

1) Individual Standards and Mixed Working Standards

Nine individual monohydroxylated PAH standard solutions were prepared by dissolving weighted amounts of target analytes in ethanol. The mixed working standards containing nine analytes were prepared from serial dilution of the individual stock solutions in 40 % (v/v) ethanol.

2) Calibration Standards

The calibration standards were prepared from serial dilution of the working standards in 40 % (v/v) ethanol. The calibration standards were aliquot into glass vials, capped and kept frozen until use.

3) Working Standard Solution of ^{13}C-labeled Standard Mix and Internal Spiking Solution (ISS)

Combine individual ^{13}C-labeled internal standard (I.S.) stock solutions (90 ng/µL) to generate the working internal standard solution (WSI). Homogenize the mixture.

Transfer 1 mL of WSI into a 500-mL volumetric flask. Dilute the solution with HPLC grade water to prepare internal spiking solution (ISS). Aliquot into amber 4-mL standard vials, cap and keep frozen until use.

d. Preparation of Quality Control Materials

1) Quality Control (QC) Materials

Prepare quality control materials by spiking a known amount of native standard mixture (in acetonitrile) into 2000 mL of an anonymous filtered urine pool. Homogenize the QC solutions for at least 3 h. Aliquot into 4-mL vials and store at -70 °C until use.

2) Proficiency Testing Material (PT)

Prepare proficiency testing materials by spiking 100 mL of an anonymous urine pool (filtered) with a known amount of working standard solution to achieve the target concentration (preferably different from those of the QCs). Prepare at least three urine pools at levels within the linear range of the method.
After spiking the urine pool with a known amount of working standard solution, homogenize the PT solutions overnight for equilibration. Then, aliquot the PT solutions into 16 x 100 mm test tubes (2 mL in each tube). PT samples are then randomized by an external PT administrator, labeled by external lab technicians, and stored at -70 °C until use.

e. Other Equipment, Materials, and Supplies

Materials / supplies and sources, or their equivalent, used during the development, validation, and application of this method are listed below.

- Incubator ovens (Fisher Scientific, INHECO)
- pH meter (Corning)
- Microbalance (Mettler-Toledo)
- Rotary suspension mixer (Glas-Col)
- Stirring/heating plates (Corning)
- Miscellaneous glassware (Pyrex, Kimax, Wheaton, Corning)
- Repeater Plus Pipette (Eppendorf)
- Electronic and Manual Pipettes (Rainin)
- Maxi-mix Vortex mixer (Barnstead International)
- Amber screw top vials of various volumes (Supelco, Inc)
- 96-Well plates (Axygen, Eppendorf)
- SBS Format Reservoirs (Seahorse Bioscience)
- Sample Tubes (Fluidx)

- Oasis WAX On-Line SPE Cartridges for Symbiosis and Prospekt 2 Systems (Merck KGaA, Darmstadt, Germany)
- Chromolith HighResolution RP-18 endcapped HPLC Column 100×4.6 mm (Merck KGaA, Darmstadt, Germany)
- Chromolith HighResolution RP-18 endcapped Guard Column 5×4.6 mm (Merck KGaA, Darmstadt, Germany)

f. Instrumentation

The sample preparation procedure can be fully automated on a Staccato® Automated System (Perkin Elmer Co.) with the following components:

- Sciclone G3/G3T
- Fluidx CESD-24PRO decapper
- Fluidx XTR-96-Cryo 2D barcode reader
- Turntable/1D barcode reader
- Hettich Rotanta 460 centrifuge
- ThermoScientific ALPS3000 sealer
- IVD Inheco Incubator Shaker DWP (4)
- Mitsubishi robotic arm

The analyses are performed on an iChrom Symbiosis online SPE system, coupled with an Agilent 1260 HPLC and AB Sciex 5500 or 6500 MS operated under negative electrospray ionization mode.

1) Online SPE

The SPE tubing and the valve switching system is used in concurrent SPE/HPLC mode controlled by the SparkLink software. The method uses both left and right cartridge clamps, the four switching valves, the high pressure dispenser, and the autosampler. The left clamp, the left clamp valve (LCV), and left integrated Stream Switching (ISS1) are used for SPE clean-up while the right clamp, the right clamp valve (RCV) and right integrated Stream Switching (ISS2) are used for the HPLC elution.

The SPE run of each sample starts with the conditioning of an Oasis WAX online cartridge with HPLC-grade acetonitrile (4 mL) and 0.1% formic acid (2 mL). Afterward, 50-500 µL of the sample (~ 50 µL of urine in 500 µL sample solution) is injected into the 1 mL sample loop and loaded onto the SPE column using 1.5 mL 0.1% formic acid. Next, the SPE column is washed with 1.5 mL Acetonitrile/Methanol/Water (1/1/2, v/v/v). The duration of the SPE procedure (including injection time) is approximately 11 min. Before starting the clean-up of the next sample, the cartridge containing the extracted analytes is transferred by a robotic gripper from the left clamp into the right clamp. While the right clamp is used for analyte elution and HPLC-MS/MS acquisition, the left clamp could be used for the clean-up of the next sample.

2) HPLC Configuration

After online SPE, the extract is loaded onto a Chromolith HighResolution RP-18 endcapped Guard Column (5×4.6 mm) with 350 µL methanol at a flow rate of 100 µL/min. The HPLC gradient (Table 4) starts with 1% methanol (with 0.1 mM ammonium fluoride) as mobile phase B at 500 µL/min for the first 3.5 minutes to focus the analytes onto the guard column. After the first 3.5 minutes, the eluent from the guard column is connected to the waste. Afterwards, the valve switches and the eluent from the guard column connects to a Chromolith column.

Column and guard column:

- o Chromolith HighResolution RP-18 endcapped HPLC Column 100×4.6 mm (Merck KGaA, Darmstadt, Germany)
- o Chromolith HighResolution RP-18 endcapped Guard Column 5×4.6 mm (Merck KGaA, Darmstadt, Germany)

HPLC Mobile Phase:

- o Mobile Phase A: 0.1 mM ammonium fluoride in Water
- o Mobile Phase B: 0.1 mM ammonium fluoride in Methanol

Table 4: HPLC Gradient.

Time (min)	Flow Rate (µL/min)	A (%)	B (%)
0.0	500	99.0	1.0
3.5	500	99.0	1.0
3.9	500	40.0	60.0
4.3	500	40.0	60.0
5.0	800	38.0	62.0
18.0	800	32.0	68.0
19.5	800	30.0	70.0
20.0	1000	15.0	85.0
21.0	1000	15.0	85.0
22.0	1000	10.0	90.0
24.0	1000	5.0	95.0
24.5	1000	5.0	95.0
24.6	1000	99.0	1.0
27.0	1000	99.0	1.0

3) Tandem Mass Spectrometer (MS/MS) Configuration

Detection of the target analytes is conducted on the AB Sciex tandem mass spectrometer in the negative electrospray ionization mode (Table 5).

Table 5. Representative Mass spectrometric parameters*

	Precursor ion (m/z)	Product ion (m/z)	Dwell Time (ms)	DP (volts)	CE (volts)
2-NAP	143	115	25	-120	-34
2-NAP2**	143	41	25	-260	-34
2-NAP-IS	149	115	25	-120	-34
1-NAP	143	115	25	-120	-34
1-NAP2**	143	41	25	-260	-34
1-NAP-IS	149	115	25	-120	-34
3-FLU	181	180	25	-100	-26
3-FLU-IS	187	186	25	-100	-26
2-FLU	181	180	25	-100	-34
2-FLU-IS	187	186	25	-100	-34
2-3PHE***	193	165	25	-150	-41
2-3PHE-IS***	199	171	25	-150	-41
1-PHE	193	165	25	-100	-38
1-PHE-IS	197	168	25	-100	-38
1-PYR	217	189	25	-100	-45
1-PYR-IS	223	195	25	-100	-45

* Actual parameters are optimized for different instruments.
** 1-NAP2 and 2-NAP2 results may be used when concentrations are ~20-200 ng/mL.
******* 2-PHE and 3-PHE are measured together (2-3PHE).

7. Calibration and Calibration Verification

a. Tuning and Calibration of Mass Spectrometer

The AB Sciex MS/MS is calibrated and tuned at least once per year using a polypropylene glycol (PPG) solution according to the instructions contained in the operator’s manual.

b. Creation of Calibration Curve

1) Calibration data

At least five calibration standards are analyzed with every analytical run. Calibration curves are generated by plotting the analyte area ratios (i.e., analyte area/internal standard area) against the native analyte concentrations through linear regression analysis with 1/X weight.

2) Evaluation of Curve Statistics

The R-squared value of the curve must be ≥0.95. Linearity of the standard curve must extend over the entire calibration range. Samples with concentrations of a given analyte exceeding the highest point in the calibration curve are reanalyzed using less urine volume.

c. Calibration Verification

1) Calibration verification is not required by the manufacturer. However, it should be performed after any substantive changes in the method or instrumentation (e.g., new internal standard, change in instrumentation), which may lead to changes in instrument response, have occurred.

2) All calibration verification runs and results shall be appropriately documented.

3) According to the updated Clinical Laboratory Improvement Amendments (CLIA) regulations from 2003 (http://www.cms.gov/Regulations-and-Guidance/Legislation/CLIA/downloads/6065bk.pdf), the requirement for calibration verification is met if the test system's calibration procedure includes three or more levels of calibration material, and includes a low, mid, and high value, and is performed at least once every six months.

4) All of the conditions above are met with the calibration procedures for this method. Therefore, no additional calibration verification is required by CLIA.

8. Procedure Operation Instructions; Calculations; Interpretation of Results

a. Sample Preparation

Sample preparation can be performed manually or on a Staccato® Automated System.

1) Enzymatic Hydrolysis

- Allow calibrators (CS), quality control samples (QCs), synthetic urine, and urine samples to thaw and reach room temperature.
- Aliquot 100 μL of specimens into a vial or plate well.
- Add 20 μL of ascorbic acid solution (12.5 mg/mL) to each vial or well.
- Add 50 μL of ISS.
- Add 50 μL of 1M sodium acetate buffer (pH 5.5) containing β-glucuronidase/arylsulfatase enzyme from *Helix pomatia* (10mg enzyme/1mL buffer) to all aliquots and mix well.
- Cap or seal all samples and incubate overnight (~18 hours) at ~37°C.

2) Centrifugation and sample transfer

After overnight enzymatic hydrolysis, add at least 175 µL methanol (total volume = 220+175 μL) to all samples and mix well to precipitate the enzyme. Cap or seal all samples and centrifuge for ~15 minutes at 3000 – 5000 rpm. Transfer 200 µL of the supernatant layer to a new sample container containing 350 µL of deionized H_2O and mix well. Transfer the rest of the supernatant layer (around 150 μL) to a second new sample container containing 350 µL of deionized H_2O, mix well and store it as backup. The backup container may be used for LC-MS analysis, and will be discarded after a month.

b. Instrument and software setup for the online SPE-HPLC-MS/MS

1) Preliminary System Setup and Performance Check

The ABSciex 5500/6500 TripleQuad mass spectrometer is calibrated and tuned periodically using positive and negative polypropylene glycol (PPG) solution provided by the manufacturer. The instrument sensitivity is checked before each analytical run by injecting the instrument test solution.

2) Runsheet and Batch Setup

A typical analytical run consists of 11 calibration standards, 3 blanks, 2 QCLs, 2 QCHs and 78 study samples.

Create a runsheet using a MS Excel runsheet template. In the **Excel** runsheet, two batch files, one for the Analyst software and one for the Sparklink software, are generated for each analytical run. The two batch files are saved as text files (.txt or .csv) to be imported to the Analyst software and Sparklink software, respectively.

3) Online SPE-HPLC-MS/MS Analysis Procedure

- Check the basic instrument functions and settings according to the manufacturer's instructions.
- Check the instrument performance by running an instrument test sample.
- In the **SparkLink** software, create a new runtable and import the Sparklink batch text or excel file created from the batch runsheet. The runtable will be automatically populated with the method, sample name, vial position, injection volume and SPE cartridge position. Make sure the correct vial position and SPE cartridge are used. Click the "start" icon on the top of the runtable.
- In the **Analyst** software, create a new batch file, then import the Analytst batch text file and the batch table will be populated with sample name, sample ID, and data file name. Make sure the correct acquisition method and quantitation method are selected. Go to the quantitation tab, and make sure all the levels of the calibration curve are filled in. Go to submit tab. Highlight the rows of samples to run and click "submit" on the top right corner. All samples on the Queue Manager should be in "waiting."
- Click "Start" on the runtable in **SparkLink** and the online SPE will start with the first sample. Then submit the batch in Analyst and click "run." Once the sample clean-up finishes, the cartridge in the left clamp will be moved by the gripper to the right clamp for elution, and the mass spectrometer will promptly start the data collection.

c. Processing of Data

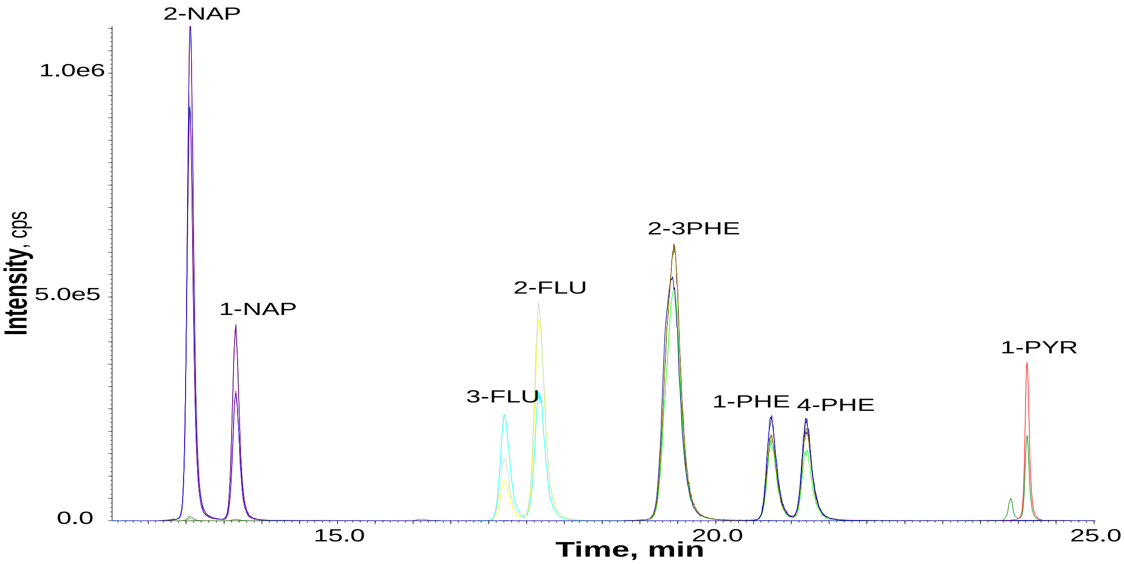

Figure 1. An example chromatogram of the standard (level 6).

An example HPLC chromatogram is given in Figure 1. Process the data using the Analyst, MultiQuant or Indigo software.

After peak integration is reviewed, go to the calibration curve page. Review the calibration curve for each analyte and confirm that R^2>0.95.

Export the results table as a text file (.txt), and place a copy of this result text file on the CDC shared network drive. Run the DLS SAS program to check the batch QCs.

d. Replacement and Periodic Maintenance of Key Components

The instrumentation used is serviced according to the manufacturers' guidance included in the instruments manuals or based on the recommendations of experienced analysts/operators after following appropriate procedures to determine that the instruments perform adequately for the intended purposes of the method.

9. Reportable Range of Results

The linear range of the standard calibration curve and the method limit of detection (LOD) determine the highest and lowest reportable concentrations for the target analytes. However, urine samples with analyte concentrations exceeding the highest reportable limit may be re-extracted using a smaller volume (e.g., 50 µL or 2 fold dilution) and re-analyzed so that the result is in the reportable range. Generally, samples with extremely high values can be diluted up to 100 times.

a. Linearity Limits

Calibration curves constructed with the analytical standards are linear for all analytes through the range of concentrations evaluated (Table 6). The linear range is up to 200 ng/mL for 1-NAP and 2-NAP (using their secondary ions) and up to 25 ng/mL for

the remaining analytes. Urine samples whose OH-PAH concentrations exceed these ranges must be diluted and re-analyzed using a smaller sample size (up to 100 times dilution).

b. Limit of Detection

The limit of detection (LOD) for each analyte is presented in Table 6.

Table 6. Limits of detection (LOD) and linearity limits.

Analyte	LOD (ng/mL)	Upper linearity limit (ng/mL)
1-NAP*	0.06	200
2-NAP*	0.09	200
2-FLU	0.008	25
3-FLU	0.008	10
1-PHE	0.009	10
2-3PHE**	0.01	20
1-PYR	0.07	10

* Upper limits for 1-NAP and 2-NAP are achieved by using the first transition using the ABSciex 6500 and the secondary ion using the ABSciex 5500, “1-NAP2” and “2-NAP2.”
** 2-PHE and 3-PHE are measured together.

c. Precision

The precision of the method is reflected in the variance of quality control samples analyzed over time. The mean concentrations and coefficients of variation (CV) of QC samples are listed in Table 7. These QC samples were analyzed over 6 weeks using two online SPE-HPLC-/MS/MS instruments.

Table 7. Mean, standard deviation, and CV for QC samples. The parameters are QC pool specific.

Analyte	QCL				QCH			
	Mean (ng/mL)	Between Day SD (ng/mL)	Within day SD (ng/mL)	CV%	Mean (ng/mL)	Between Day SD (ng/mL)	Within day SD (ng/mL)	CV%
1-NAP	1.17	0.09	0.05	6.9%	7.65	0.62	0.46	6.9%
2-NAP	1.53	0.14	0.08	8.0%	8.84	0.65	0.41	6.6%
2-FLU	0.26	0.02	0.01	8.6%	1.12	0.10	0.07	8.0%
3-FLU	0.29	0.02	0.01	6.5%	1.20	0.08	0.06	5.2%
1-PHE	0.18	0.03	0.01	13.7%	0.70	0.09	0.05	12.2%
2-3PHE	0.39	0.05	0.02	11.2%	1.47	0.15	0.09	9.3%
1-PYR	0.37	0.05	0.03	12.8%	0.73	0.11	0.08	13.8%

d. Analytical Specificity

The analyte peaks are located in well-defined regions of the chromatogram with no visible interferences and low background.

e. Accuracy

The accuracy of this method was evaluated by analyzing two NIST Standard Reference Materials (SRMs) and by comparing the results obtained to their certified concentrations for the 9 OH-PAHs (Table 8). The smoker SRM 3672 and the non-smoker SRM 3673 were analyzed 4 times in 2 different runs. Averages of the measured concentrations are given in Table 8.

Table 8. Measured concentrations (ng/mL) using this method in comparison to the certified concentrations in two NIST SRMs (5)

Analyte	SRM 3672 Smoker urine			SRM 3673 non-Smoker urine		
	This method	NIST Certified	Accuracy	This method	NIST Certified	Accuracy
2-NAP	8.182	8.730	93.7%	1.438	1.345	106.9%
1-NAP	36.853	34.400	107.1%	216.064	211.000	102.4%
3-FLU	0.414	0.428	96.8%	0.044	0.039	112.0%
2-FLU	0.720	0.870	82.8%	0.090	0.107	83.7%
2-3PHE	0.201	0.209	96.1%	0.055	0.053	103.0%
1-PHE	0.115	0.136	84.3%	0.048	0.049	98.4%
1-PYR	0.177	0.173	102.2%	0.051	0.030	169.7%*

*: <3LOD.

The accuracy of the method was further assessed by repeated analyses (n=7) of three spiking concentrations in synthetic urine (Table 9).

Table 9. Accuracy

Analyte	Level -1		Level -2		Level -3	
	Spiked (ng/mL)	Accuracy	Spiked (ng/mL)	Accuracy	Spiked (ng/mL)	Accuracy
2-NAP	0.708	112.8%	3.845	99.7%	19.226	99.7%
1-NAP	0.705	113.3%	3.826	95.3%	19.130	98.7%
3-FLU	0.168	107.2%	0.915	98.6%	4.573	98.1%
2-FLU	0.176	107.4%	0.956	99.3%	4.778	100.3%
2-3PHE	0.357	113.1%	1.937	99.4%	9.684	101.9%
1-PHE	0.175	109.8%	0.951	99.4%	4.753	101.2%
1-PYR	0.179	105.5%	0.972	93.6%	4.860	101.8%

10. Quality Assessment and Proficiency Testing

a. Quality Assessment

Daily experimental checks are made on the stability of the analytical system. Two QCLs and two QCHs are prepared and placed randomly on each run. The concentrations of the two QCH and two QCL are averaged to obtain one measurement of QCH and QCL for each batch.

b. Quality Control Procedures

1) Individual Sample Quality Checks

Each individual sample will be subjected to a number of quality checks:

- Auto integrations must be reviewed and integrated manually if needed
- The relative retention time (RRT) of each analyte, if detectable, in relation to its respective IS must be within 0.995 – 1.005 (e.g. 0.12 min difference between the native and labeled 1-PYR). Check integration if the RRT falls out of the range. If a peak is present with its retention time out of the limit in relation to its IS retention time, an analytical interference may prevent the correct measurement of the target analyte, and the result for that analyte is coded as non-reportable (NR).

2) Establishing QC limits

Quality control limits are established by characterizing assay precision with repeated analyses of the QC pools. Different variables are included in the analysis (e.g. multiple analysts and instruments) to capture realistic assay variation over time. The mean, standard deviation (within day and between day), coefficient of variation, and confidence limits are calculated from this QC characterization data set. Individual quality control charts for the

characterization runs are created, examined, and quality control limits are used to verify assay precision and accuracy on a daily basis. QC characterization statistics for OH-PAH analytes are listed in Table 7. Characterization statistics are pool specific.

3) Quality Control Evaluation

After the completion of a run, the quality control concentrations are evaluated to determine if the run is "in control." The quality control rules apply to the average of the two replicates of each of the QC pools. The quality control results are evaluated according to a multi-rule QC check (6), and standard criteria for run rejection based on statistical probabilities are used to declare a run either in-control or out-of-control.

Two QC pools *per run* with two or more QC results *per pool*

A) If both QC run means are within $2S_m$ limits and individual results are within $2S_i$ limits, then accept the run.

B) If 1 of the 2 QC run means is outside a $2S_m$ limit - reject run if:

a) Extreme Outlier – Run mean is beyond the characterization mean +/- $4S_m$
b) 3S Rule - Run mean is outside a $3S_m$ limit
c) 2S Rule - Both run means are outside the same $2S_m$ limit
d) 10 X-bar Rule – Current and previous 9 run means are on same side of the characterization mean

C) If one of the 4 QC individual results is outside a $2S_i$ limit - reject run if:

a) Extreme Outlier – One individual result is beyond the characterization mean +/- $4S_i$
b) R 4S Rule – Within-run ranges for all pools in the same run exceed $4S_w$ (i.e., 95% range limit). Note: Since runs have multiple results per pool for 2 pools, the R 4S rule is applied within runs only.

Abbreviations:

S_i = Standard deviation of individual results (the limits are not shown on the chart unless run results are actually single measurements).
S_m = Standard deviation of the run means (the limits are shown on the chart).
S_w = Within-run standard deviation (the limits are not shown on the chart).

If the QC result for an analyte is declared "out of control", the results of that analyte for all study samples analyzed during that run are invalid for reporting.

c. Proficiency Testing (PT)

The in-house proficiency testing (PT) scheme for this method is administered by an in-house PT coordinator. PT samples are prepared in-house by spiking a

known amount of standard into a well characterized urine pool and blind-coded by an in-house PT coordinator. PT samples are analyzed twice a year using the same method described for unknown samples.

In addition to the in-house PT program, a minimum of once per year, we also analyze two reference urine samples fortified with 1-NAP, 2-NAP and 1-PYR (as of 2015) as part of the German External Quality Assessment Scheme (G-EQUAS). G-EQUAS is organized and managed by the Institute and Outpatient Clinic for Occupational, Social and Environmental Medicine of the University of Erlangen-Nuremberg (Erlangen, Germany). The program, evaluation, and certification are based on the guidelines of the German Federal Medical Council (http://www.g-equas.de/).

All proficiency results shall be appropriately documented. If the assay fails proficiency testing, then the sample preparation and instrumentation are thoroughly examined to identify and correct the source of assay error.

11. Remedial Action if Calibration or QC Systems Fail to Meet Acceptable Criteria

If the calibration or QC systems fail to meet acceptable criteria, suspend all operations until the source or cause of failure is identified and corrected. If the source of failure is easily identifiable, for instance, failure of the mass spectrometer or a pipetting error, the problem is immediately corrected. Otherwise, prepare fresh reagents and clean the mass spectrometer system. Before beginning another analytical run, re-analyze several QC materials (in the case of QC failure) or calibration standards (in the case of calibration failure). After re-establishing calibration or quality control, resume analytical runs. Document the QC failures, review the situation with the supervisor and/or his/her designee to determine source(s) of problem, and take measures to prevent re-occurrence of the same problem.

12. Limitations of Method, Interfering Substances and Conditions

This is an isotope dilution mass spectrometry method, widely regarded as the definitive method for the measurement of organic toxicants in human body fluids. By using tandem mass spectrometry, most analytical interferences are eliminated. However, unknown endogenous substances may interfere with the chromatographic separation of certain analytes and/or suppress instrument sensitivity, especially when the urine samples are non-fasting or may also contain many other potential substances in addition to the target analytes (e.g., smokers' urine). To overcome inadequate chromatography or analytical sensitivity, dilute the sample and re-inject ("instrument rerun"). If the instrument rerun results are not acceptable, repeat the sample preparation by using up to 100 times dilution. If the diluted analysis still results in an interference that cannot be separated chromatographically or severe suppression of the target analyte signal and/or its IS, the result for that analyte is coded as not reportable (NR).

13. Reference Ranges (Normal Values)

Reference range values for the OH-PAH metabolites, established based on the National Health and Nutrition Examination Survey (NHANES), can be found at http://www.cdc.gov/exposurereport.

14. Critical Call Results ("Panic Values")

Insufficient data exist to correlate urinary OH-PAH concentrations with serious health effects in humans. Therefore, no established "critical call" values exist. Test results in this laboratory are reported in support of epidemiological studies, not for clinical assessments.

15. Specimen Storage and Handling During Testing

Urine specimens may reach and maintain ambient temperature during analysis. The urine extracts are stored in a sample collection plate (when prepared using the Staccato automated method) at -70 ^{o}C after analysis. CDC's unpublished data suggest that these extracts are stable for at least one month.

16. Alternate Methods for Performing Test or Storing Specimens if Test System Fails

A GC-MS/MS method is available on site if necessary for measuring these PAH metabolites in urine (3). Furthermore, aliquoting and spiking of the urine can also be done manually if the Staccato automated system fails. Upon system failures, urine extracts can be refrigerated for up to a week until the analytical system is restored to functionality.

17. Test Result Reporting System; Protocol for Reporting Critical Calls (if Applicable)

Study subject data can be reported both in concentration units (e.g., µg/L, ng/mL, ng/L, pg/mL) and/or adjusted based on creatinine excretion (e.g., µg/g creatinine).

a. The data from each analytical run are initially processed and reviewed by the laboratory supervisor, his/her designee or Quality Control officer to check sample Quality Control parameters (e.g., recovery, relative retention time, blank levels, calibration curve). The supervisor and/or his/her designee can provide feedback to the analyst and request confirmation of the data as needed.
b. The Quality Control officer reviews each analytical run, identifies the quality control samples within each analytical run, and determines whether the analytical run is performed under acceptable control conditions.
c. One of the Division statisticians reviews and approves the quality control charts pertinent to the results being reported.
d. If the quality control data are acceptable, the laboratory supervisor or his/her designee generates a memorandum to the Branch Chief, and a letter reporting the analytical results to the person(s) who requested the analyses to be signed by the Division Director.
e. The data are sent (generally electronically by e-mail) to the person(s) who made the initial request.

Final hard copies of correspondence are maintained in the office of the Branch Chief and/or his/her designee and/or with the quality control officer.

18. Transfer or Referral of Specimens; Procedures for Specimen Accountability and Tracking

Following successful completion of analysis, the urine must be returned to storage at -70 °C in case reanalysis is required. Urine samples shall be retained until valid results have been obtained and reported and sufficient time has passed for review of the results. Residual urine may be returned to the study PI or properly discarded upon completion of the project.

Standard record keeping (e.g., database, notebooks, data files) is used to track specimens. Specimens can be transferred or referred to other DLS Branch laboratories or, if required, to other laboratories. Transfer is normally carried out through the DLS Samples Logistic Group. Specimens may also be stored at CDC specimen handling and storage facility (CASPIR).

19. Summary Statistics and QC Graphs

See following pages.

2013-2014 Summary Statistics and QC Chart for 1-Hydroxynapthalene (1-Naphthol) (ng/L)

Lot	N	Start Date	End Date	Mean	Standard Deviation	Coefficient of Variation
QC6_201207a	4	16JAN14	22JAN14	1181.38	44.79	3.8
QC6_201207b	77	16JAN14	05AUG14	1139.10	52.95	4.6
QC5_201207a	4	16JAN14	22JAN14	766.25	25.38	3.3
QC5_201207b	77	16JAN14	05AUG14	755.16	36.90	4.9
QCL14F	5	01AUG14	27AUG14	1112.20	31.33	2.8
QCM14F	5	01AUG14	27AUG14	3496.60	159.83	4.6
4108	37	05NOV15	02MAR16	7562.03	169.04	2.2
4107	37	05NOV15	02MAR16	1175.95	41.60	3.5

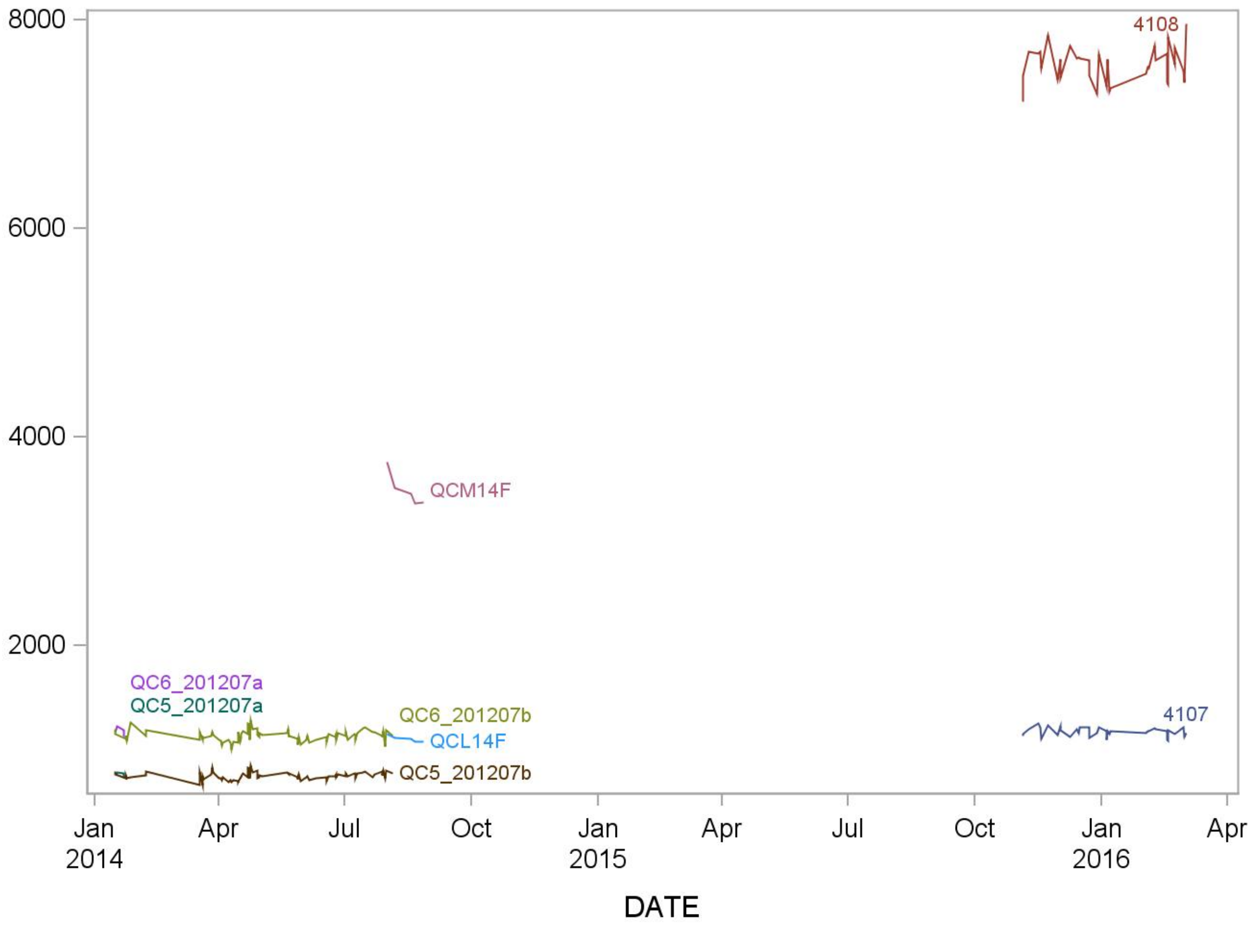

2013-2014 Summary Statistics and QC Chart for 1-Hydroxyphenanthrene (ng/L)

Lot	N	Start Date	End Date	Mean	Standard Deviation	Coefficient of Variation
QC6_201207a	4	16JAN14	22JAN14	728.00	9.93	1.4
QC6_201207b	79	16JAN14	31JUL14	687.87	23.01	3.3
QC5_201207a	4	16JAN14	22JAN14	291.38	3.38	1.2
QC5_201207b	79	16JAN14	31JUL14	279.09	10.57	3.8
QCL14F	4	01AUG14	27AUG14	216.50	13.15	6.1
QCM14F	4	01AUG14	27AUG14	638.88	41.98	6.6
4108	36	05NOV15	02MAR16	714.15	25.64	3.6
4107	36	05NOV15	02MAR16	182.29	10.00	5.5

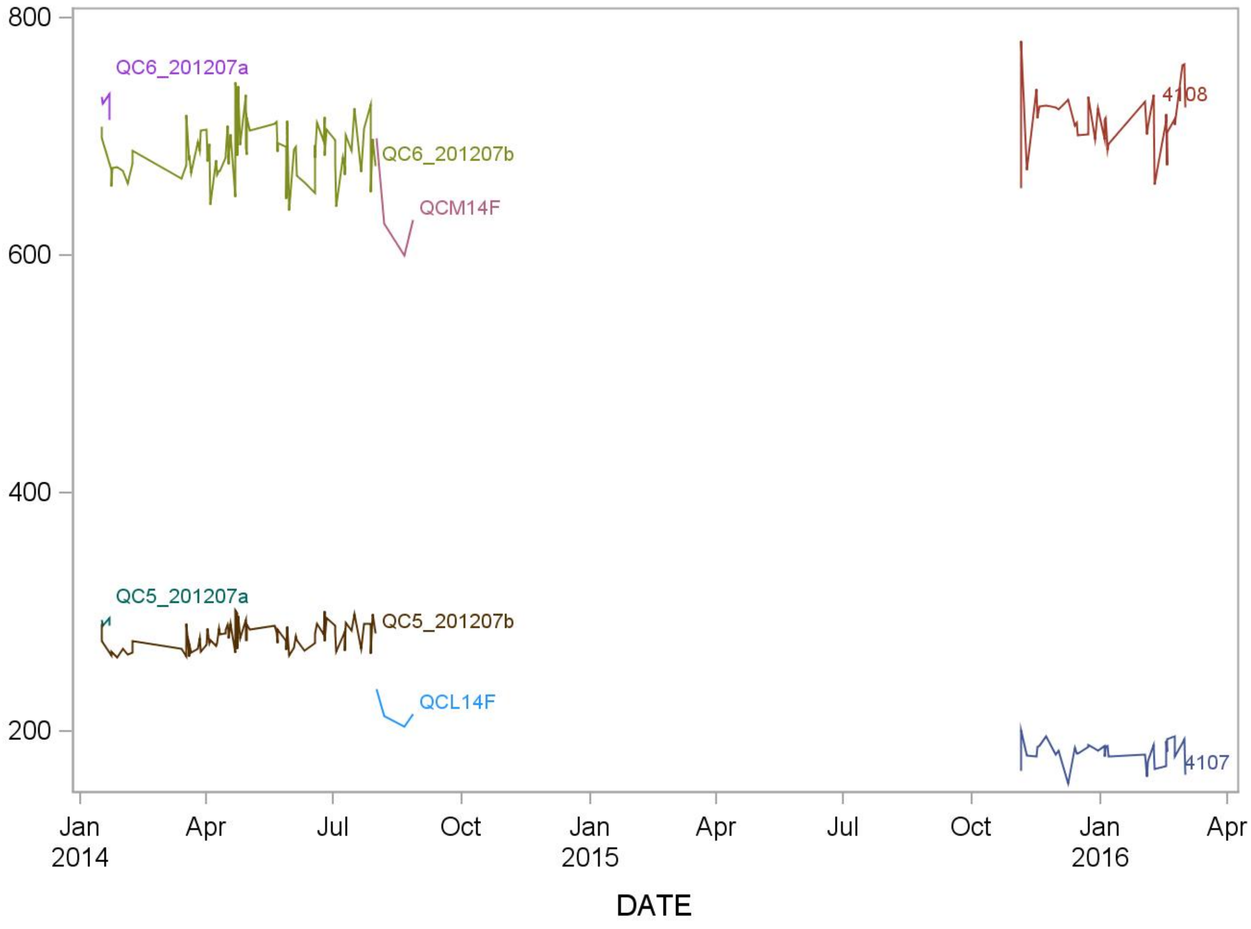

2013-2014 Summary Statistics and QC Chart for 1-Hydroxypyrene (ng/L)

Lot	N	Start Date	End Date	Mean	Standard Deviation	Coefficient of Variation
QC6_201207a	4	17JAN14	05FEB14	745.25	34.30	4.6
QC5_201207a	4	17JAN14	05FEB14	266.63	16.36	6.1
QC6_201207b	78	23JAN14	31JUL14	737.99	43.16	5.8
QC5_201207b	78	23JAN14	31JUL14	269.15	16.99	6.3
QCL14F	4	01AUG14	27AUG14	450.63	37.60	8.3
QCM14F	4	01AUG14	27AUG14	739.13	86.28	11.7
4108	36	05NOV15	02MAR16	910.57	59.41	6.5
4107	36	05NOV15	02MAR16	440.47	29.95	6.8

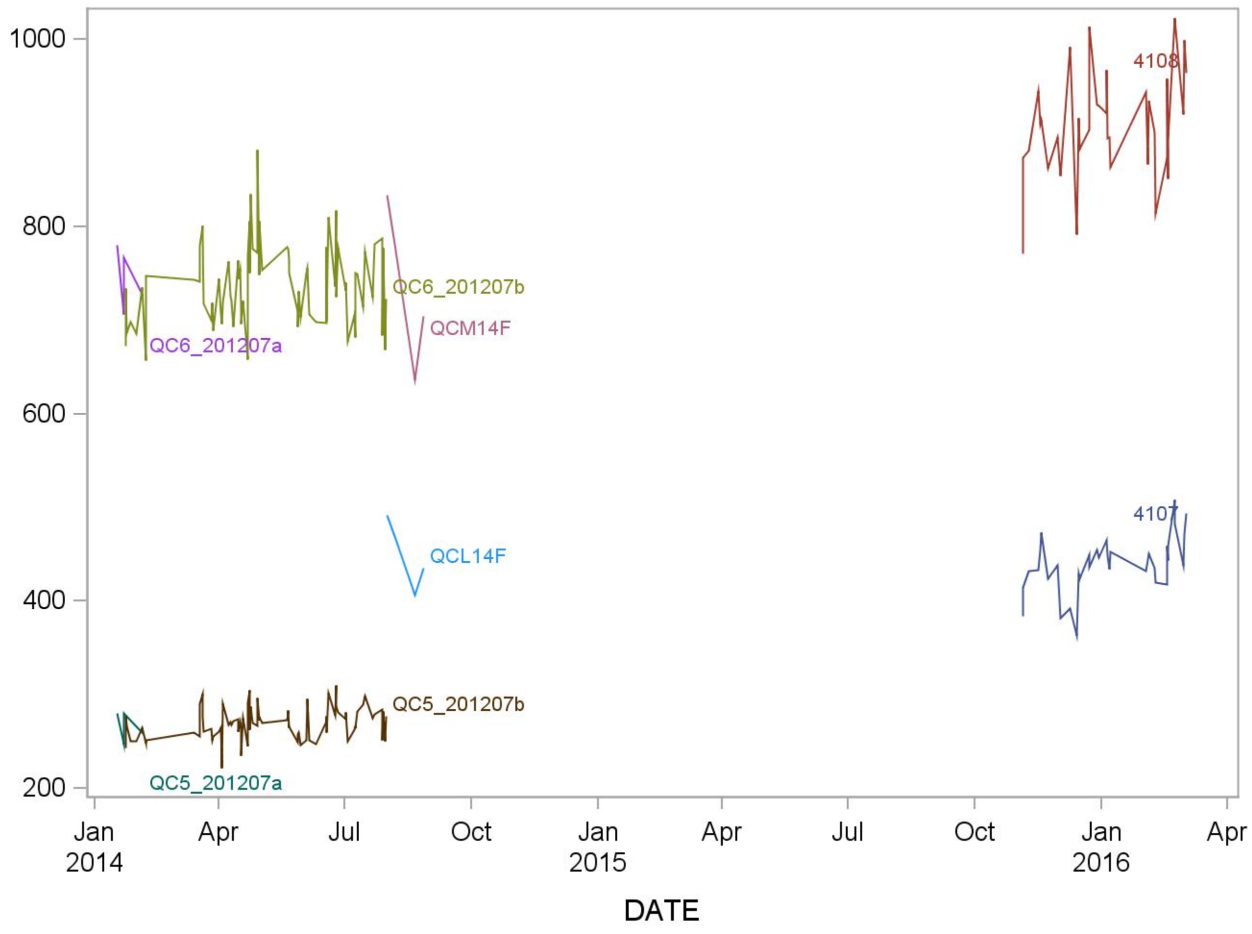

2013-2014 Summary Statistics and QC Chart for 2 & 3-Hydroxyphenanthrene (ng/L)

Lot	N	Start Date	End Date	Mean	Standard Deviation	Coefficient of Variation
4108	36	05NOV15	02MAR16	1393.06	59.25	4.3
4107	36	05NOV15	02MAR16	361.88	14.75	4.1

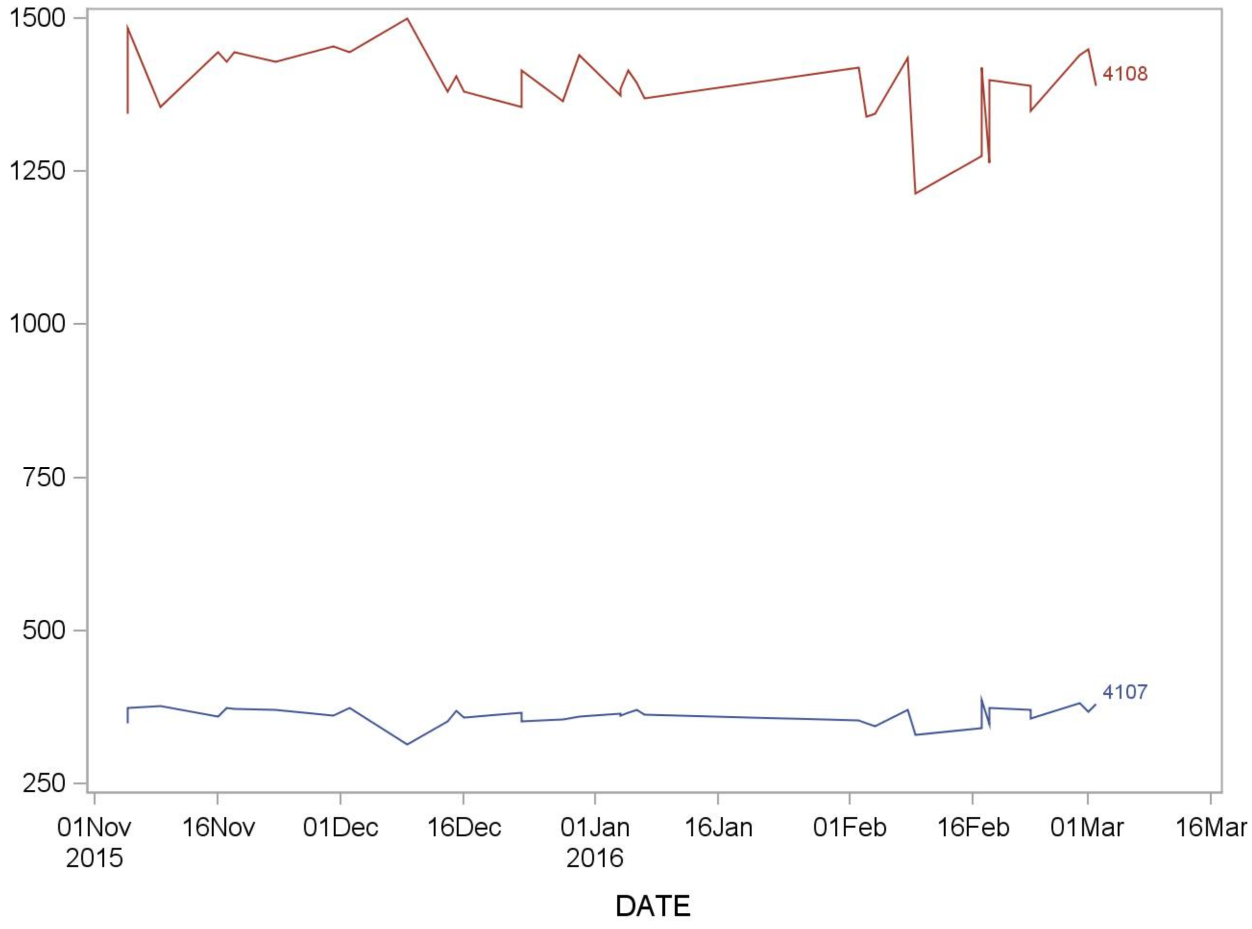

2013-2014 Summary Statistics and QC Chart for 2-Hydroxyfluorene (ng/L)

Lot	N	Start Date	End Date	Mean	Standard Deviation	Coefficient of Variation
QC6_201207a	4	16JAN14	22JAN14	615.38	15.35	2.5
QC6_201207b	80	16JAN14	31JUL14	565.26	27.33	4.8
QC5_201207a	4	16JAN14	22JAN14	301.13	10.74	3.6
QC5_201207b	80	16JAN14	31JUL14	283.73	13.37	4.7
QCL14F	4	01AUG14	27AUG14	254.25	12.25	4.8
QCM14F	4	01AUG14	27AUG14	679.00	42.43	6.2
4108	36	05NOV15	02MAR16	1042.44	22.51	2.2
4107	36	05NOV15	02MAR16	239.83	6.23	2.6

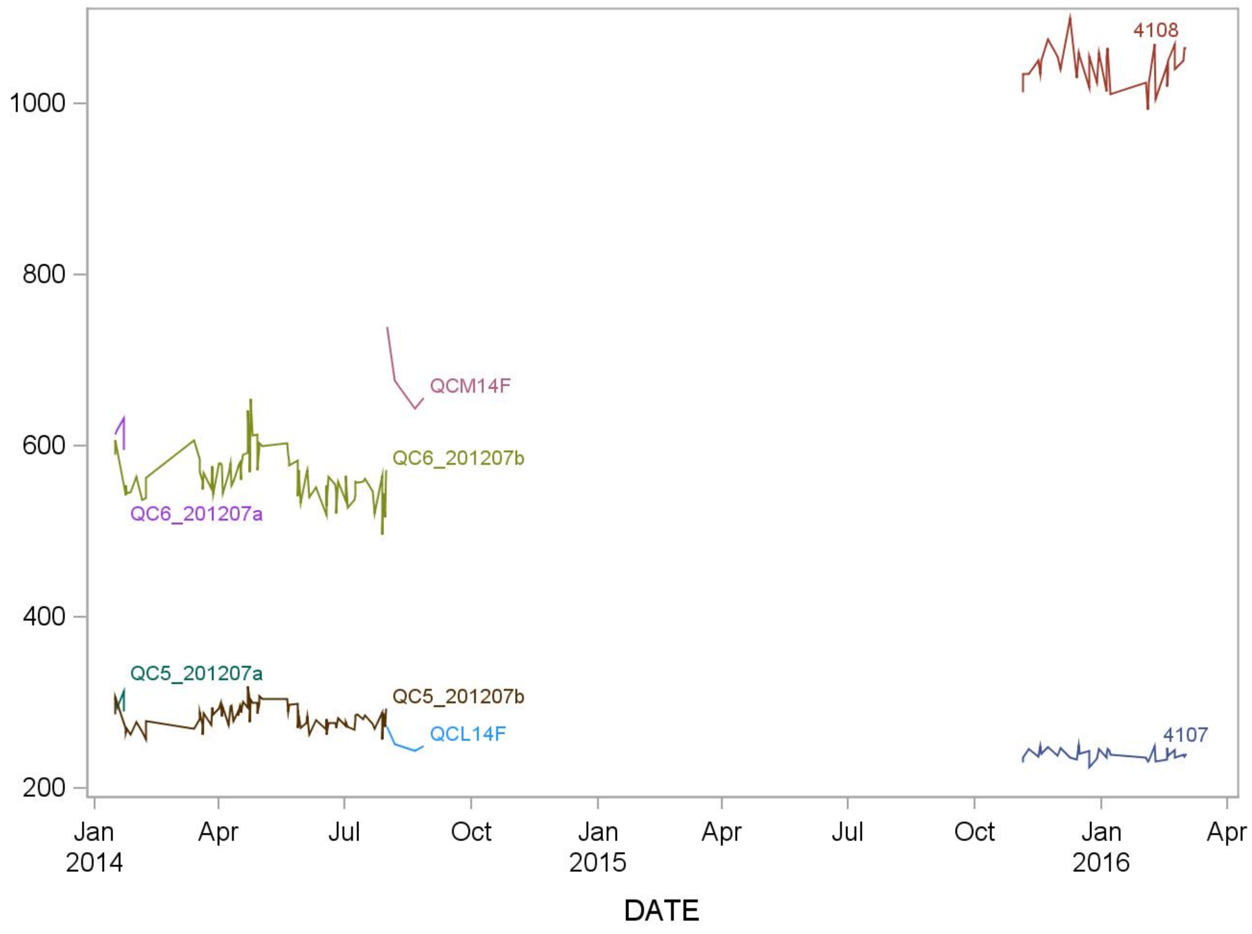

2013-2014 Summary Statistics and QC Chart for 2-Hydroxynapthalene (2-Naphthol) (ng/L)

Lot	N	Start Date	End Date	Mean	Standard Deviation	Coefficient of Variation
QC6_201207a	4	16JAN14	22JAN14	2178.63	136.27	6.3
QC6_201207b	80	16JAN14	31JUL14	2028.63	88.38	4.4
QC5_201207a	4	16JAN14	22JAN14	1704.50	97.11	5.7
QC5_201207b	80	16JAN14	31JUL14	1607.63	70.57	4.4
QCL14F	4	01AUG14	27AUG14	1350.13	56.62	4.2
QCM14F	4	01AUG14	27AUG14	3866.00	214.16	5.5
4108	37	05NOV15	02MAR16	8447.97	233.43	2.8
4107	37	05NOV15	02MAR16	1447.03	51.62	3.6

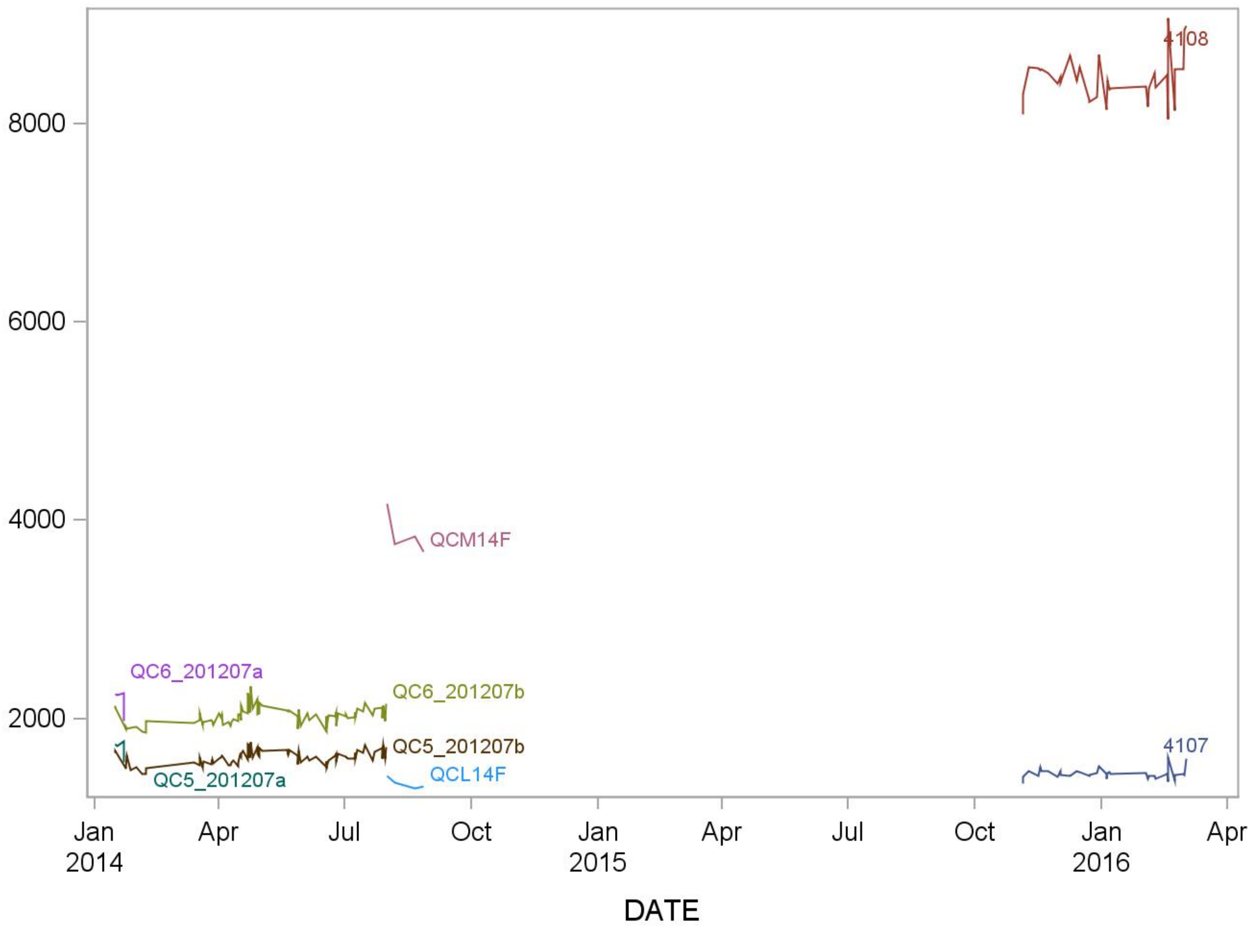

2013-2014 Summary Statistics and QC Chart for 3-Hydroxyfluorene (ng/L)

Lot	N	Start Date	End Date	Mean	Standard Deviation	Coefficient of Variation
QC6_201207a	4	16JAN14	22JAN14	628.88	28.32	4.5
QC6_201207b	80	16JAN14	31JUL14	586.10	41.78	7.1
QC5_201207a	4	16JAN14	22JAN14	243.25	15.25	6.3
QC5_201207b	80	16JAN14	31JUL14	229.97	17.31	7.5
QCL14F	4	01AUG14	27AUG14	254.63	20.93	8.2
QCM14F	4	01AUG14	27AUG14	736.25	62.72	8.5
4108	36	05NOV15	02MAR16	1137.36	25.54	2.2
4107	36	05NOV15	02MAR16	268.71	4.88	1.8

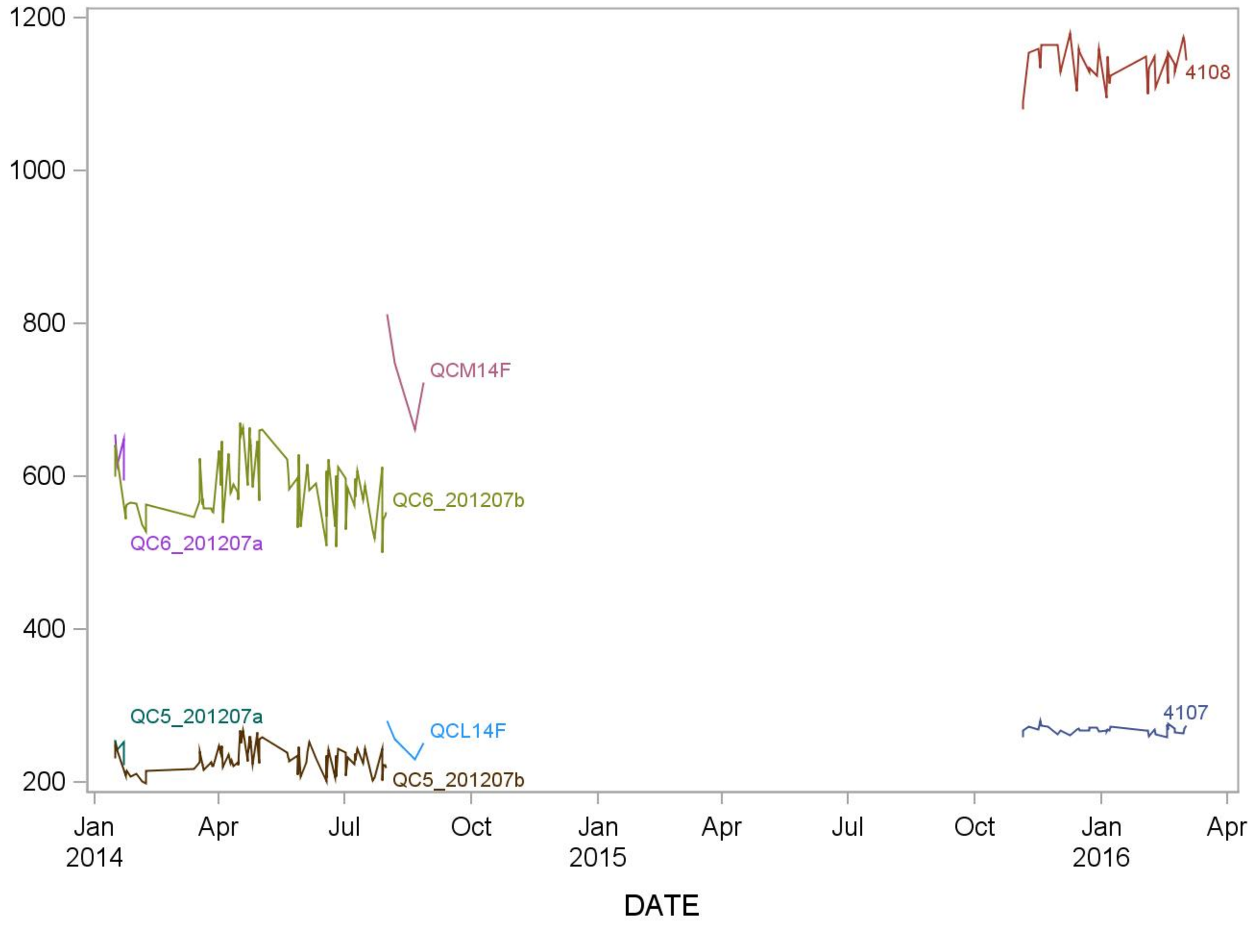

20. References

1. IARC. 2010, vol 92 IARC Monographs on the Evaluation of Carcinogenic Risks to Humans. Some Non-heterocyclic Polycyclic Aromatic Hydrocarbons and Some Related Exposures. http://monographs.iarc.fr/ENG/Monographs/vol92/mono92.pdf.

2. ATSDR (1995) Toxicological Profile for Polycyclic Aromatic Hydrocarbons. Agency for Toxic Substances and Disease Registry. Atlanta, GA. http://www.atsdr.cdc.gov/toxprofiles/tp69.pdf.

3. Li Z, Romanoff LC, Trinidad DA, Pittman EN, Hilton D, Hubbard K, Carmichael H, Parker J, Calafat AM, Sjödin A. 2014 "Quantification of Twenty-one Metabolites of Methylnaphthalenes and Polycyclic Aromatic Hydrocarbons in Human Urine", Anal Bioanal Chem 406(13):3119-29.

4. Li Z, Romanoff LC, Trinidad D, Hussain N, Porter EN, Jones RS, Patterson Jr DG, Sjodin A. 2006. "Measurement of Urinary Mono-Hydroxylated Polycyclic Aromatic Hydrocarbons Using Automated Liquid-Liquid Extraction and Isotope Dilution Gas Chromatography/High Resolution Mass Spectrometry". Anal Chem 78:5744-5751.

5. Schantz MM, Benner BA Jr, Heckert NA, Sander LC, Sharpless KE, Vander Pol SS, Vasquez Y, Villegas M, Wise SA, Alwis KU, Blount BC, Calafat AM, Li Z, Silva MJ, Ye X, Gaudreau É, Patterson DG Jr, Sjödin A. 2015. "Development of urine standard reference materials for metabolites of organic chemicals including polycyclic aromatic hydrocarbons, phthalates, phenols, parabens, and volatile organic compounds", Anal Bioanal Chem, DOI 10.1007/s00216-014-8441-02015 Feb 5. [Epub ahead of print]

6. Caudill SP, Schleicher RL, Pirkle JL. 2008. Multi-rule quality control for the age-related eye disease study. Stat Med 27:4094-4106.

Appendix 1

Body mass index (BMI): a measure of weight adjusted for height, calculated as weight in kilograms divided by the square of height in meters (kg/m^2).

Smoking status: (1) never: < 100 cigarettes per lifetime; (2) former: > 100 cigarettes per lifetime yet no longer smoke; (3) now: > 100 cigarettes per lifetime and smoke at times or daily.

Drinking status: (1) never: never drunk in life; (2) former: previous alcohol consumption but no alcohol consumption in the last 12 months; (3) mild: drinker who not met the other criteria; (4) moderate: 2-3 drinks per day for females, 3-4 drinks per day for males, or 2-5 binge drinks per month; (5) heavy: three or more drinks per day for females, four or more drinks per day for males, or more than 5 binge drinks per month.

Cardiovascular disease (CVDs): the diagnosis was confirmed when any of the following symptoms occurred: coronary heart disease; congestive heart failure; heart attack; stroke; angina.

Chronic kidney disease (CKD): ACR (urine albumin-creatinine ratio) >= 30mg/mmol or eGFR (estimated glomerular filtration rate) <= (60ml/min/1.73 m^2).

Hypertension: (1) systolic >= 140 mm hg; (2) diastolic >= 90 mm hg; (3) number of tests for diagnose hypertension >= 3. Any of the above conditions is sufficient.

Hyperlipidemia: (1) hypertriglyceridemia: TG (triglyceride) >= 150 mg/dL; (2) hypercholesterolemia: TC (total cholesterol) >= 200 mg/dL[5.18mmol/L]; LDL >= 130 mg/dL[3.37mmol/L]; HDL < 40mg/dL [1.04 mmol/L] (male), 50mg/dL [1.30 mmol/L] (female); (3) used antihyperlipidemic agents. Meet any one of these three criteria can be included.

Appendix 2 Concentration distribution of polycyclic aromatic hydrocarbons (PAHs) by quartile grouping in the National Health and Nutrition Examination Survey (NHANES) 2005–2014 (n = 2,573).

Quartile	1-Hydroxynaphthalene(ng/l)	Frequency	Percentage
Q1	[33.9,698]	648	25.18%
Q2	(698,1629]	639	24.83%
Q3	(1629,6226.6]	643	24.99%
Q4	(6226.6,27066310.1]	643	24.99%
Quartile	2-Hydroxynaphthalene(ng/l)	Frequency	Percentage
Q1	[117.1,1701.6]	644	25.03%
Q2	(1701.6,3980.4]	643	24.99%
Q3	(3980.4,9565.6]	643	24.99%
Q4	(9565.6,560484.3]	643	24.99%
Quartile	3-Hydroxyfluorene(ng/l)	Frequency	Percentage
Q1	[3.5,32]	646	25.28%
Q2	(32,66.7]	632	24.74%
Q3	(66.7,202]	639	25.01%
Q4	(202,7886.8]	638	24.97%
Quartile	2-Hydroxyfluorene(ng/l)	Frequency	Percentage
Q1	[8.7,96]	643	25.03%
Q2	(96,196]	642	24.99%
Q3	(196,502]	642	24.99%
Q4	(502,21271.3]	642	24.99%
Quartile	1-Hydroxyphenanthrene(ng/l)	Frequency	Percentage
Q1	[6.4,63]	649	25.22%
Q2	(63,120.9]	639	24.83%
Q3	(120.9,225.8]	642	24.95%
Q4	(225.8,7019]	643	24.99%
Quartile	1-Hydroxypyrene(ng/l)	Frequency	Percentage
Q1	[3.5,49.5]	759	29.73%
Q2	(49.5,97]	523	20.49%
Q3	(97,209.5]	633	24.79%
Q4	(209.5,9970.5]	638	24.99%

Appendix 3 |nteraction analysis of common confounders with miscarriage risk in NHANES 2005–2014 (n = 2,573).

Character	95% CI	*p* for interaction	95% CI	*p* for interaction	95% CI	*p* for interaction
Interactive effect	BMI and abortion		age and abortion		smoke and abortion	
Smoke		0.36		0.06		
Former	0.024(-0.016,0.065)		-0.017(-0.034,0.000)			
Never	0.002(-0.009,0.013)		-0.001(-0.005,0.003)			
Now	0.009(-0.007,0.025)		0.003(-0.006,0.012)			
Race		0.60		0.51		0.02
Mexican American	0.01(-0.008,0.029)		-0.003(-0.008,0.002)		0.53	
Non-Hispanic Black	0.008(-0.009,0.024)		0.005(-0.002,0.013)		0.19	
Non-Hispanic White	0.025(-0.025,0.075)		-0.005(-0.024,0.014)		0.54	
Other Hispanic	-0.003(-0.018,0.012)		-0.007(-0.014,0.001)		0.71	
Other Race	-0.003(-0.035,0.030)		0(-0.017,0.017)		0.01	
Alcohol		0.99		0.47		0.28
Never	0.011(-0.016,0.038)		-0.004(-0.015,0.006)		0.30	
Former	0.007(-0.013,0.027)		0(-0.006,0.006)		0.30	
Mild	0.01(-0.006,0.026)		-0.003(-0.010,0.003)		0.24	
Moderate	0.01(-0.015,0.034)		-0.008(-0.017,0.002)		0.32	
Heavy	0.004(-0.021,0.028)		0.005(-0.006,0.015)		0.74	
CVD		0.86		0.19		0.09
No	0.008(-0.006,0.022)		-0.003(-0.007,0.001)		0.76	
Yes	0.01(-0.010,0.030)		-0.013(-0.028,0.002)		0.03	
CKD		0.65		0.54		0.18
No	0.008(-0.008,0.025)		-0.003(-0.007,0.001)		0.64	
Yes	0.014(-0.004,0.031)		-0.006(-0.016,0.004)		0.95	

BMI, body mass index; PIR, poverty income ratio; CVD, cardiovascular diseases; CKD, chronic kidney disease.

© 2025 Liu X. et al.
